# Membrane Transport Modulates the pH-Regulated Feedback of an Enzyme Reaction Confined within Lipid Vesicles

**DOI:** 10.1021/acsnano.4c13048

**Published:** 2025-03-03

**Authors:** Darcey Ridgway-Brown, Anna S. Leathard, Oliver France, Stephen P. Muench, Michael E. Webb, Lars J. C. Jeuken, Peter J. F. Henderson, Annette F. Taylor, Paul A. Beales

**Affiliations:** † School of Chemistry, University of Leeds, Leeds LS2 9JT, U.K.; ‡ Astbury Centre for Structural Molecular Biology, University of Leeds, Leeds LS2 9JT, U.K.; § Chemical and Biological Engineering, University of Sheffield, Sheffield S1 3JD, U.K.; ∥ School of Biomedical Sciences, Faculty of Biological Sciences, University of Leeds, Leeds LS2 9JT, U.K.; ⊥ Leiden Institute of Chemistry, University Leiden, PO Box 9502, 2300 RA Leiden, The Netherlands; # School of Chemistry and Chemical Engineering, University of Southampton, Southampton SO17 1BJ, U.K.

**Keywords:** enzymes, membrane, confinement, permeability, pH, vesicles, urease

## Abstract

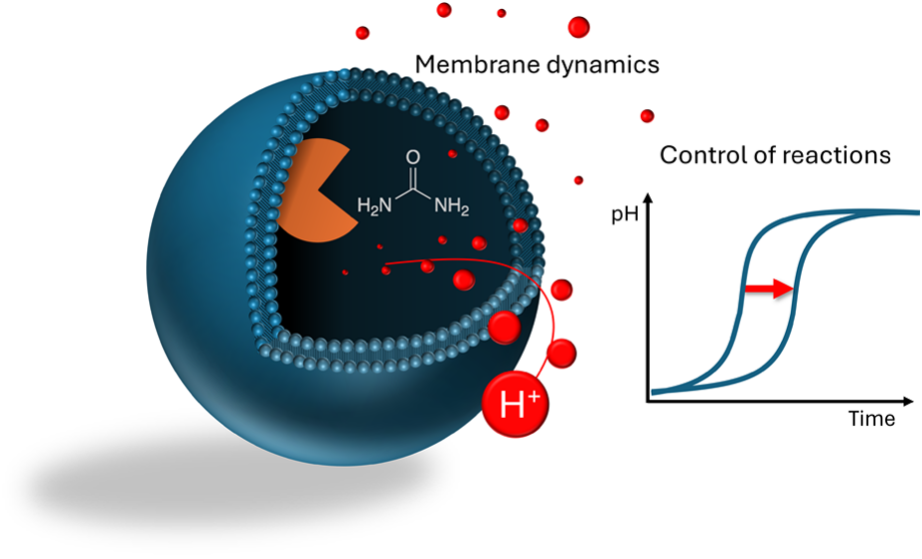

Understanding ion transport dynamics in reactive vesicles is pivotal for exploring biological and chemical processes and essential for designing synthetic cells. In this work, we investigate how proton transport and membrane potential regulate pH dynamics in an autocatalytic enzyme reaction within lipid vesicles. Combining experimental and numerical methods, we demonstrate that compartmentalization within lipid membranes accelerates internal reactions, attributed to protection from the external acidic environment. In experiments, we explored how proton movement significantly impacts internal reactions by changing bilayer thickness, adding ion transporters, and varying buffers. Numerical investigations incorporated electrical membrane potential and capacitance into a kinetic model of the process, elucidating the mechanisms that dictate the control of reaction time observed in the experiment, driven by both electrical and chemical potential gradients. These findings establish a framework for controlling pH clock reactions via membrane changes and targeted manipulation of proton movement, which could aid in the design of synthetic cells with precise, controlled functionalities.

## Introduction

In nature, most enzymes are membrane-bound or compartmentalized, where their local environment influences their catalytic activity. This phenomenon explains why enzyme behavior often changes once purified and isolated.^1^ Membranes serve as highly functionalized, selective barriers. Therefore, within each membrane-bound compartment of a cell, a specialized intracellular space is cultivated through the regulated transfer of chemical and physical information.^2^ As a result, *in situ* enzyme reaction rates depend on the permeability of substrates, which is controlled both spatially and temporally through membranes.^3^ Additionally, more complex underlying mechanisms can regulate enzyme reaction dynamics within membrane-bound confinement. For example, intracellular pH can alter the charge and structure of macromolecules such as enzymes, thereby regulating their activity.^4^ Furthermore, the movement of charged species across a membrane can induce a membrane potential that drives the movement of protons across the membrane, influencing pH and further impacting enzyme activity.^5^ Proton gradients are a key biological feature in bioenergetic systems, as seen in mitochondria for ATP synthesis via chemiosmosis, where respiration-driven proton pumps create an electrochemical proton gradient, negative and alkaline inside.^6,7^ Controlled proton leaks across membranes are also significant. For instance, proton leaks contribute to thermogenesis by generating heat in brown adipose tissue or modulate the production of reactive oxygen species (ROS) by preventing excessive proton gradient buildup and protecting cells from oxidative stress.^7^ Additionally, to regulate intracellular pH through electroneutral exchange, cells can employ cation/proton antiporters, such as Na^+^/H^+^ exchangers.^7^

Consequently, the interplay between proton transport, membrane permeability, and enzyme activity is fundamental to the maintenance of cellular homeostasis and function. To gain a deeper insight into membrane compartmentalization and electrochemical gradients, more accurately mimic cellular processes, and ensure programmable behavior for biomedical applications, it is essential to elucidate how confinement, membrane transport and pH can influence enzyme behavior.

Lipid vesicles, formed from self-assembling phospholipid bilayers, are indispensable tools in synthetic biology for studying and replicating cell-like behavior in confined volumes. Biocompatible and mimicking natural cell membranes, they are ideal for creating minimal interfaces in artificial cells.^8,9^ Lipid vesicles are extensively used in biomedical applications where they encapsulate bioactive molecules for applications in drug delivery, biosensors, and nanoreactors.^10−13^ In these settings, understanding how pH changes can control, and influence functionality is crucial. The design of nonlinear dynamics in pH-feedback enzyme systems has shown promising applications in drug release, biosensors, nanomotors and hydrogel formation.^14−21^ The selective permeability of lipid vesicles can regulate their functionality, making it an important property to understand and control. In this work, we investigate a minimal cell system by encapsulating an autocatalytic enzyme reaction in lipid vesicles. Through a combination of experimental studies and numerical simulations, we demonstrate the role of confinement and proton transport on the internalized reactions.

**Figure 1:**
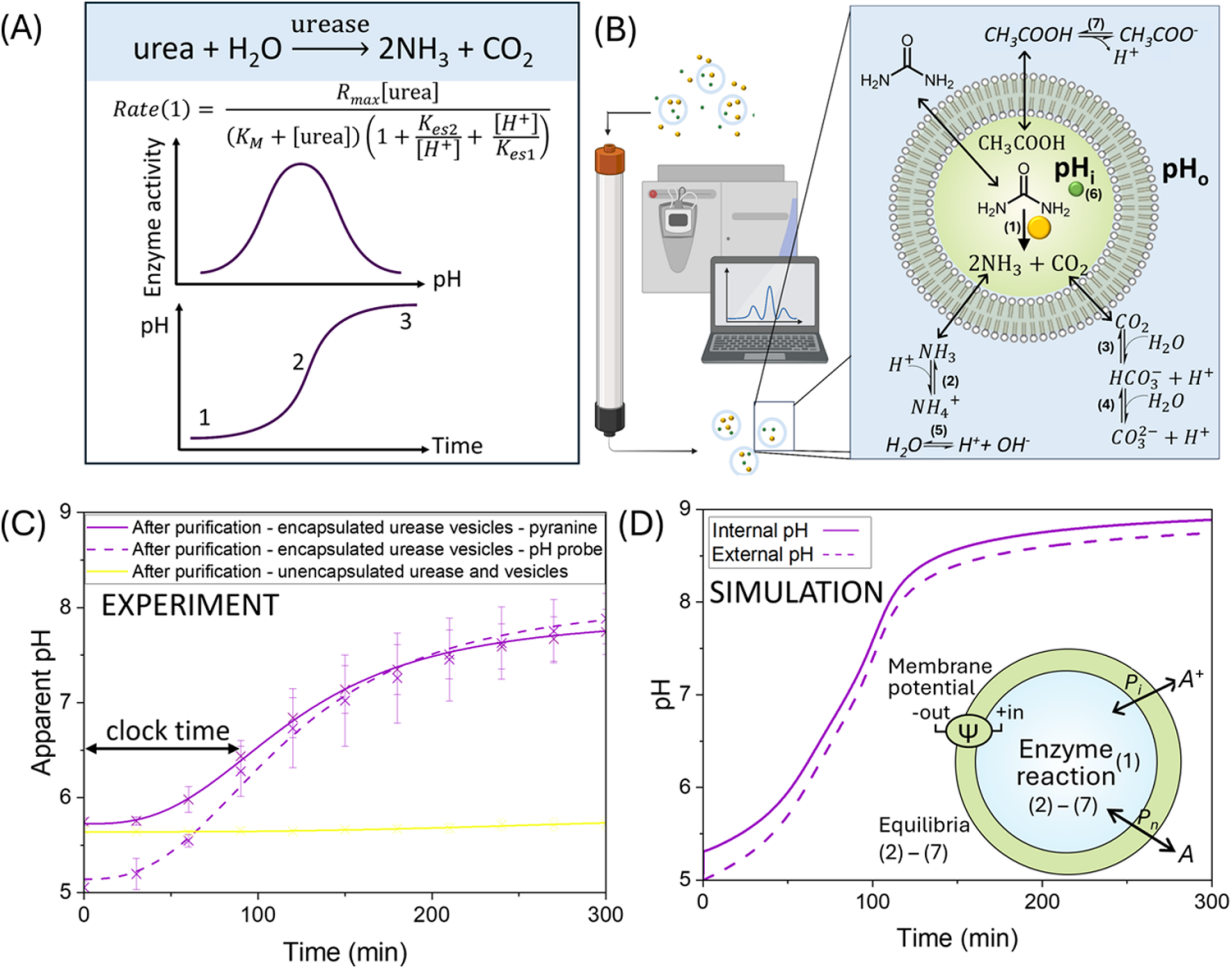
Preparation and characterization of urease encapsulated in synthetic vesicles and validation of our theoretical model. (A) Sketch of the bell-shaped enzyme activity described by a pH-dependent Michaelis–Menten rate eq 1, with acid binding constants *K*_es1_, *K*_es2_ and maximum rate *R*_max_ (top panel), leading to a pH–time curve of the urea–urease reaction (bottom panel). (B) Schematic diagram of the purification of vesicles where external urease and pyranine are removed using size exclusion chromatography and reaction with urease (yellow) and pyranine (green) confined to a vesicle (pH_i_), urea in the outer solution (pH_o_) and the relevant equilibria (created with BioRender.com). (C) Verifying the purification of vesicles (purple, solid) using the apparent pH change calculated from the fluorescent intensity ratio (450/405 nm) of pyranine after addition of urea to urease vesicles (in acetate buffer with NaCl) with external pH measured using a pH probe (purple, dashed), and control with urease-free vesicles (yellow), where external 20 μM urease was removed by size exclusion chromatography. (D) Simulation of a pH clock in vesicles, showing internal (solid) pH_i_ and external (dashed) pH_o_ and inset to illustrate vesicle with transport of neutral species *A* with permeabilities *P*_n_, transport of ions *A*^+^ with permeabilities *P_i_*, enzyme reaction and equilibria. The *R*_max_ = 0.066 M/min and internal vesicle concentrations were: [acetate buffer] = 50 mM, [H^+^] = 1 × 10^–5^ M, [Na^+^] = 120 mM, [Cl^–^] = 70 mM and external concentrations were: [urea] = 25 mM, [acetate buffer] = 25 mM, [H^+^] = 1 × 10^–5^ M, [Na^+^] = 50 mM, [Cl^–^] = 35 mM. For all other parameters, see methods section.

Urease, like most enzymes, has a pH-dependent activity with a maximum rate around pH 7. It catalyzes the hydrolysis of urea into ammonia and carbon dioxide (Figure 1A).^22−24^ If the reaction is initiated at a low pH (Figure 1A1), the pH will increase as the reaction progresses. This increase in pH enhances urease activity, causing the reaction to accelerate through its optimal pH (Figure 1A2), after which the reaction rate will decelerate (Figure 1A3). This product-catalyzed feedback mechanism through the production of ammonia results in a sigmoidal profile known as a pH clock reaction (Figure 1A).^25^ Owing to its pH-dependence and the significant differences between membrane permeability of reactive species and products, such as positively charged H^+^ ions and neutral molecules like urea and ammonia, urease-encapsulated lipid vesicles are an ideal minimal system to explore the role of membranes, confinement, pH change and permeability.^26^

The urea–urease system has been spatially confined to investigate the role of diffusion and induced spatiotemporal control on pH-dependent feedback.^14,27−31^ Selective permeability and confinement were explored both computationally and experimentally using urease-encapsulated nanoscale and microscale vesicles by Miele et al., who demonstrated collective synchronized behavior between vesicles due to fast ammonia transport.^31^ Urease has also been partitioned in liquid condensate droplets at similar metabolic densities to cells, where it appears to produce steady pH gradients leading to self-generated flow, drawing similarities with cellular processes such as cytoplasmic streaming.^30^ Additionally, spatially distributed urease was able to support propagating fronts through diffusion of ammonia, its autocatalytic species, converting pH from low to high.^29^ Itatani et al. demonstrated that the urease-esterase reaction network can generate temporal pH waveforms in giant unilamellar vesicles, combining experimental and modeling approaches.^32^ Numerically, differential transport of H^+^ and urea has also been shown to produce autonomous pH oscillations in the urea–urease system; however, this is yet to be seen experimentally.^31,33^ Van Hest et al. encapsulated urease in pH sensitive polymersomes, where membrane permeability could switch between an ON/OFF state dependent on pH.^27^ Permeability has also been controlled using light by incorporating photosensitive molecules into urease polymersomes.^28^ These findings collectively demonstrate the potential for developing synthetic cells with tunable behavior through membrane transport.

While simulation studies have examined how the membrane capacitance influences the flux of charged species,^5,34,35^ these processes have not been incorporated into models of reactive vesicles. Consequently, there is a gap in understanding how these interactions control reaction dynamics within cell-like compartments.

Here we provide significant insights into the urease reaction encapsulated inside nanoscale lipid vesicles by elucidating the roles of confinement, membrane potential and transport in regulating the reaction dynamics.^31^ Utilizing numerical simulations to deepen our understanding of experimental observations, we further develop an ordinary differential equation (ODE) kinetic model of the urease reaction inside lipid vesicles to incorporate membrane potential and the effects of facilitated ion transport by specific solutes into a reactive vesicle for the first time. The membrane-specific regulatory mechanisms we uncover provide knowledge that is not only essential for fundamental insights into cellular function but also paves the way for designing adaptable, controllable, and application-driven bioengineered systems with a broad range of uses.

## Results and Discussion

### Construction of Urease Vesicles

Initially we re-establish our experimental and theoretical models for urease reactions confined within lipid vesicles based on previous work from our groups.^31^ We encapsulated 20 μM urease (11 mg/mL of type III, Sigma-Aldrich, Supporting Information (SI) 1.1) in 50 mM sodium acetate buffer at pH 5 (100 mM ionic strength with NaCl) within 164 ± 3 nm diameter DOPC lipid vesicles (from DLS (SI 1.2.4)). To monitor the reaction, we also encapsulated the fluorescent pH indicator pyranine (50 μM) and measured the fluorescent intensity ratio (450/405 nm).^36^ This can be converted into apparent pH to monitor the change in pH over time inside the vesicles, due to ammonia production (SI 1.2.1). Afterward, size exclusion chromatography (SEC) was used to separate vesicles from external urease and pyranine based on size, and a solution of urease vesicles in acetate buffer was mixed with a solution of urea to initiate reaction (Figure 1B). To confirm the removal of external urease, 50 μM pyranine vesicles were made with 20 μM urease added externally before being purified using SEC. A reaction with any remaining urease was initiated with 25 mM urea. No pH change was found between the purified 50 μM pyranine vesicles and urea, confirming the sufficient removal of external urease (Figure 1C, yellow). Consequently, we can be confident that any pH change observed in our experiments is due to urease encapsulated in lipid vesicles. An encapsulation efficiency of urease in lipid vesicles was calculated as 54 ± 9% using the activity of urease (SI 1.3), i.e., the average urease concentration in the aqueous lumen of the lipid vesicles was estimated to be a maximum of 10.8 ± 1.8 μM.

In Figure 1C, we see the characteristic sigmoidal pH clock reaction profile of urease (purple, solid) in vesicles,^37^ indicative of the nonlinear reaction dynamics due to the production of ammonia influencing the pH-dependent activity of urease over time.^31^ This suggests the pH-dependent feedback of urease is still retained when encapsulated in lipid vesicles. The pH of the external solution (purple, dashed) increased more slowly than the internal pH, resulting in a proton gradient between the vesicles and the surrounding solution. To compare how each clock reaction progresses, we can determine the time to reach the average midpoint of pH switching behavior (pH 6.4. SI 1.2.2), known as the clock time (min). For example, the measured average clock time of our urease vesicles is 73.3 ± 7 min. This was performed on a fluorimeter, but in order to measure multiple conditions simultaneously, a plate reader was used for the remaining experiments.

### Modeling the Urease Vesicles

Insights were gained from an ordinary differential equation (ODE) model of vesicles and the external solution (SI 3), which describes the rate of change of species inside and outside the compartment. In Figure 1D, we see the simulated characteristic sigmoidal pH clock profile of the urease reaction in vesicles, demonstrating that the internal pH (solid) is initially higher (pH = 5.3) than the external (dashed) pH = 5, in agreement with experiments. This small difference in pH corresponds to a 50% reduction of the hydrogen ion concentration and can lead to a significant decrease in clock time, as seen in the next section. Due to uncertainty in model parameters, such as permeability coefficients and certain enzyme parameters, our simulations provide a semiquantitative approach to understanding the experiments. We aim to reproduce key experimental features and trends with a minimal model that demonstrates how membrane transport influences enzyme clock reaction behavior in lipid vesicles.

The model is based on previous work from our group on the urea–urease system.^31^ In this iteration of the model, we have incorporated membrane capacitance and membrane potential to account for their influence on the transmembrane flux of ionic species, following methodologies in the literature.^5,34,35,38^ Stochastic effects were not considered as previous work has demonstrated that population-level behavior is retained within ODE models of these systems.^33^

Inside the vesicles, the rate of change of the concentration of a species *A*_*i*_ is determined by the reaction rate terms, *f*(*A*_i_), and mass transport term *g*(*A*_i_, *A*_o_) where *A*_o_ is the concentration of the species in the external solution. Transport of neutral species (NH_3_, CO_2_, urea, acetic acid) across the vesicle membrane is driven by a concentration gradient
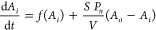
1where *f*(*A*_*i*_) contains all the relevant reaction rate and equilibria terms from (1) to (7) indicated in Figure 1B, *S* is the surface area, *V* is the vesicle volume, *P*_*n*_ is the permeability coefficient of the neutral species. Transport of ions across the membrane is driven by diffusion and migration as result of the electrical field associated with the membrane potential, Ψ. The rate of change of the ionic species is determined through reaction and mass transport using the Goldman-Hodgkin-Katz (GHK) flux equation
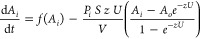
2where *P*_*i*_ is the permeability coefficient of the ionic species, *z* is the charge (valency) on ion *i*, *U* is the reduced membrane potential, defined as *U =* ΔΨ/*(k*_B_*T*/*e*). This dimensionless quantity normalizes the membrane potential by the thermal voltage, *k*_B_*T*/*e* where *k*_B_ is the Boltzmann constant, *T* is temperature and *e* is elementary charge. At room temperature (298 K), the thermal voltage is 25.69 mV. The membrane potential between inside and outside the vesicle, ΔΨ, is related to the net charge accumulated in the vesicle using a capacitor model for the membrane^39^ and is defined as follows
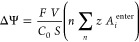
3where *F* is Faraday’s Constant, *C*_*o*_ is the membrane capacitance and *A*_*i*_^enter^ is the internal concentration of ions that have entered the lumen. We included transport of Na^+^, Cl^–^, K^+^ and H^+^ as the most important ionic species contributing to the membrane potential.

The transport of species across lipid membranes depends on multiple factors including the nature of the lipid headgroup, number of carbons in the hydrocarbon chain and the degree of saturation, and experimental conditions which may affect bilayer thickness and rigidity.^40^ Here, in the absence of literature data for the specific phospholipids used, we took values of the permeability coefficients within ranges given in the literature for neutral species with *P_n_* = 10^–4^ – 10 cm/min, small anions with *P*_i_ = 10^–9^ – 10^–6^ cm/min, and small cations with *P_i_* = 10^–7^ – 10^–12^ cm/min, and in the order of *P*_CO_2__, *P*_NH_3__, > *P*_AA_, *P*_urea_ > *P*_Cl–_ ≫ *P*_Na+_, *P*_K+_.^41^ Reported values of the permeability of protons vary greatly from 10^–8^ – 10 cm/min, reflecting possible changes in the nature of species crossing (such as H_9_O_4_^+^) and mechanism of transport under different conditions.^42^ The permeability coefficient of H^+^ is often used as a fitting parameter in experiments with phospholipid membranes; we take *P*_H+_ = 10^–3^ cm/min for DOPC. Capacitance increases with decreasing bilayer thickness and increasing salt concentration^43^ and is of the order of 1 μF/cm^2^ for phospholipid vesicles.^44,45^ We explore the effect of variations of proton permeability and capacitance on the urease clock reaction in the next section.

The rate of change of the concentration of species *A*_*o*_ in the external solution is given by
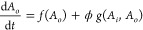
4where *f*(*A*_o_) includes the relevant equilibria terms, *g*(*A*_i_, *A*_o_) contains mass transport terms of neutral or ionic species and the vesicle volume fraction (or concentration) is given by ϕ = *NV*/*V*_*o*_, assuming *N* identical vesicles of volume *V* and *V*_*o*_ is the external volume of solution. The volume fraction accounts for the dilution of species when transferred from the nanovesicles to the outer solution. For a population of *N* identical vesicles, the total volume of vesicles (*NV*) in a 1 mL sample was calculated to be ∼5 × 10^–2^, giving, after dilution during purification and addition of urea, a vesicle volume fraction ϕ of the order of 10^–3^; here we took ϕ = 2 × 10^–3^. More details of the model parameters and assumptions are included in the Methods section and in SI 3.1, 3.4–3.5.

### Introducing Membrane Potential and Proton Influx

For an enzyme with a pH-dependent feedback mechanism, such as urease, the transport of protons across the vesicle membrane can significantly affect the reaction dynamics. Unlike the electroneutral transport of uncharged molecules like ammonia, the movement of charged ions, such as protons, along their concentration gradients induces an electrical potential and an electrical gradient. This prevents the system from reaching chemical equilibrium and fosters a new electrochemical equilibrium. Over time, this results in a nonconstant flux of charged species across a membrane, which can affect the reaction dynamics within the vesicle.

**Figure 2:**
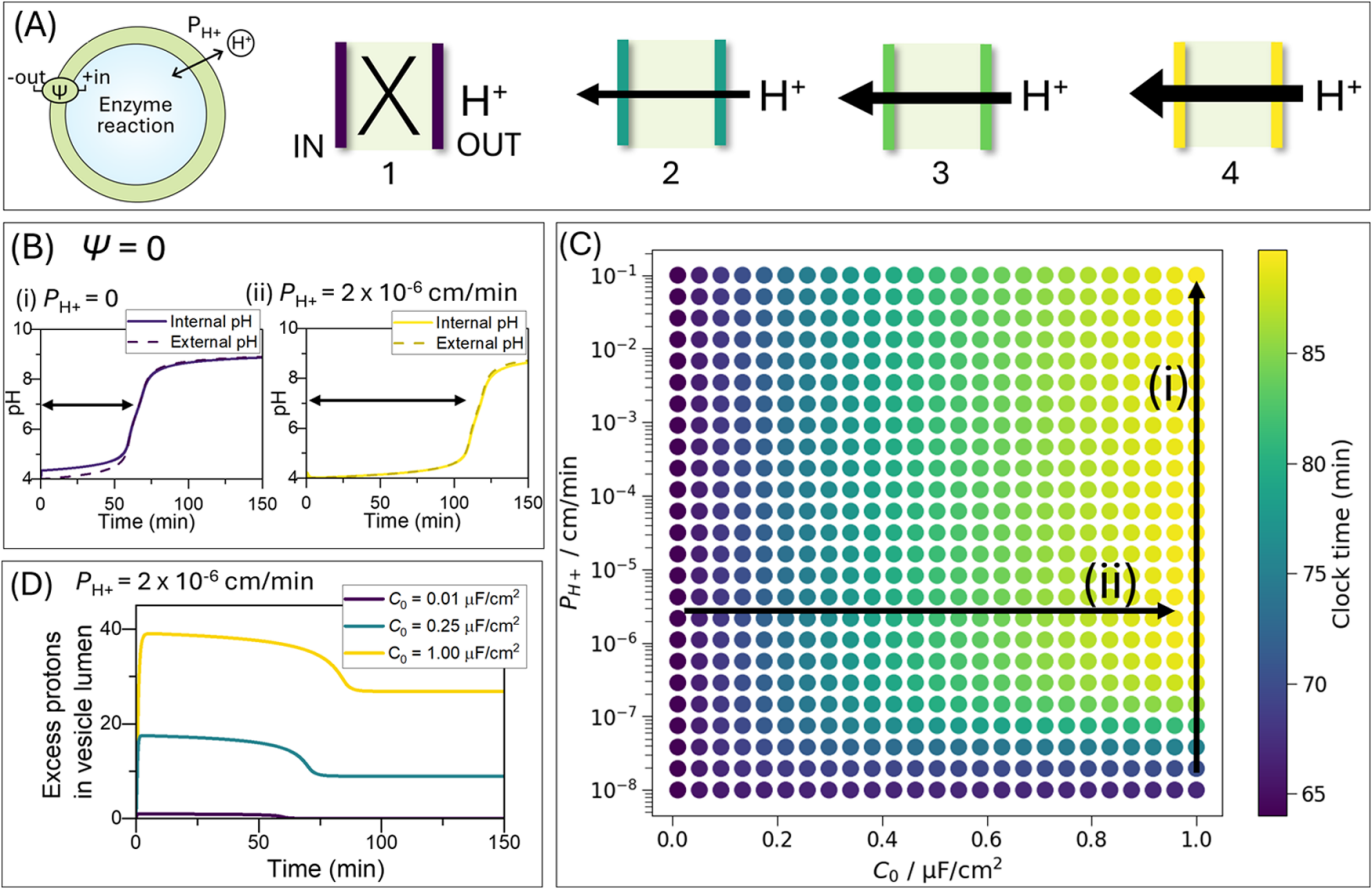
Proton influx slows the urea–urease clock reaction in simulations of lipid vesicles. Simulations were performed with enzyme rate *R*_max_ = 0.0029 M/min, internal vesicle concentrations: [H^+^] = 1 × 10^–4^ M, no buffer, and external solution: [urea] = 25 mM, [H^+^] = 1 × 10^–4^ M. For all other parameters see methods section. (A) Schematic representation of the vesicle and four membrane scenarios: (1) Proton impermeable membrane (purple); (2–4) Proton-permeable membranes, with increasing permeability or capacitance values. (B) pH traces over time inside vesicles (solid curve, pH_i_) and in the outer solution (dashed curve, pH_o_) with clock time times indicated for membrane potential fixed at zero and the reaction with a (i) proton impermeable membrane and (ii) proton permeable membrane. (C) Map of clock times (time to pH 6.4) as a function of proton permeability and membrane capacitance, with the arrows indicating: (i) constant capacitance of ∼1 μF/cm^2^, and (ii) constant proton permeability of 2 × 10^–6^ cm/min. (D) Count of excess protons in the vesicle lumen caused by transport through the membrane for capacitance values indicated and constant permeability.

To understand the influence of membrane potential on proton influx and its impact on the urea–urease clock reaction, we modeled the effects of proton transport under a range of conditions, with no buffer and protons as the only charged species allowed to cross the membrane (illustrated in Figure 2A). In Figure 2B, the clock reaction is shown for two limiting cases with the membrane potential fixed at zero. When the vesicles are impermeable to proton transport, the simulated reaction follows a typical course, initially reaching an internal pH around 4.3 before both internal and external pH levels equalize, resulting in a clock time of 64 min (Figure 2Bi). The initial pH difference between the inside and outside of the vesicle creates a driving force for proton influx, which would occur if the membrane allowed proton movement. In Figure 2Bii, permeability of protons is enabled, with membrane potential maintained at zero. In this case, the internal vesicle pH and outer solution pH are the same during the reaction and because of the lower internal pH, the clock time nearly doubles to 114 min.

Introducing membrane potential into the model allows for regulation of proton transport based on the electrochemical gradient. Proton flow is modulated by both the permeability coefficient *P*, which dictates the rate of chemical gradient-driven proton movement, and membrane capacitance *C*_*o*_, which can limit proton influx when the electrical potential increases across the membrane. Figure 2C shows the clock time as a function of *P*_H+_ and *C*_0_ with initial concentrations of [H^+^] = 1 × 10^–4^ M and [urea] = 25 mM. For these conditions, there is an increase in clock time when (i) the capacitance is held constant at 1 μF/cm^2^ while proton permeability is increased, and (ii) proton permeability is constant at 2 × 10^–6^ cm/min while capacitance is increased.

In scenarios with constant capacitance (Figure 2Ci), low proton permeability (e.g., 1 × 10^–8^ cm/min) restricts proton flux, with a 3 min increase in clock time (to 67 min) compared to no permeability. Higher proton permeability allows protons to enter the vesicle more rapidly, maintaining a lower internal pH and thus slowing down the reaction, which extends the clock times. However, when proton permeability is sufficiently high (1 × 10^–5^ cm/min), the buildup of charge across the membrane opposes further proton transport, causing the clock times to plateau despite high permeability.

In scenarios with constant permeability (Figure 2Cii), high permeability (2 × 10^–6^ cm/min) means that capacitance becomes the controlling factor in clock time. Membrane capacitance determines the amount of charge the membrane can store. Capacitance values typical of biological and artificial lipid membranes (<1 μF/cm^2^),^43,46^ result in highly restricted proton flux when the membrane potential increases. Consequently, for the initial concentrations used here, clock times show minimal changes when capacitance is increased in the model from 0 to 0.25 μF/cm^2^. This reflects the limited number of protons crossing the membrane, with a maximum excess of just 17 protons at 0.25 μF/cm^2^ (Figure 2D). At higher capacitances, the number of protons entering the vesicle increases, extending the clock time to 90 min with a maximum excess of 40 protons. As the reaction proceeds and the chemical gradient in acid is reduced, the opposing electrical gradient then drives excess protons out from the lumen.

Thus, our model demonstrates that both the permeability coefficient *P*_H+_ and capacitance *C*_0_ significantly influence proton transport, and by extension, the rate of the enzyme-catalyzed reactions within vesicles. Neglecting these electrochemical aspects may result in oversimplified models that fail to capture the nuanced dynamics of encapsulated reactions.

### Changing Membrane Thickness

To investigate the role of confinement and nonspecific membrane permeability on the encapsulated urease reaction, we changed bilayer thickness by making vesicles of different lipid compositions. Our vesicles were constructed from phosphatidylcholines (PC) with two fatty acid chains of 18-carbon chain length and a double bond on the ninth carbon of each chain (18:1 (Δ9-Cis)). We investigated the effect of decreasing the chain length by 2 and 4 carbons (16:1 (Δ9-Cis) PC and 14:1 (Δ9-Cis) PC, respectively). The encapsulation efficiency of urease (in acetate buffer with NaCl) for all lipid vesicles was calculated (SI 1.3.2) and found not to be significantly different between 18:1 (Δ9-Cis) PC and 14:1 (Δ9-Cis) PC (54 ± 9 and 66 ± 24%, respectively), allowing confidence that changes to reaction dynamics are due to membrane transport. Encapsulation efficiency of 16:1 (Δ9-Cis) PC was found to be significantly higher (94 ± 18%) than 18:1 (Δ9-Cis) PC but this did not correlate to the trend found, implying membrane thickness rather than urease encapsulation efficiency has the dominant effect on the observed behavior. Dynamic light scattering was also performed, where hydrodynamic diameters of vesicles were not significantly different between these different lipid compositions (SI 1.2.4).

**Figure 3:**
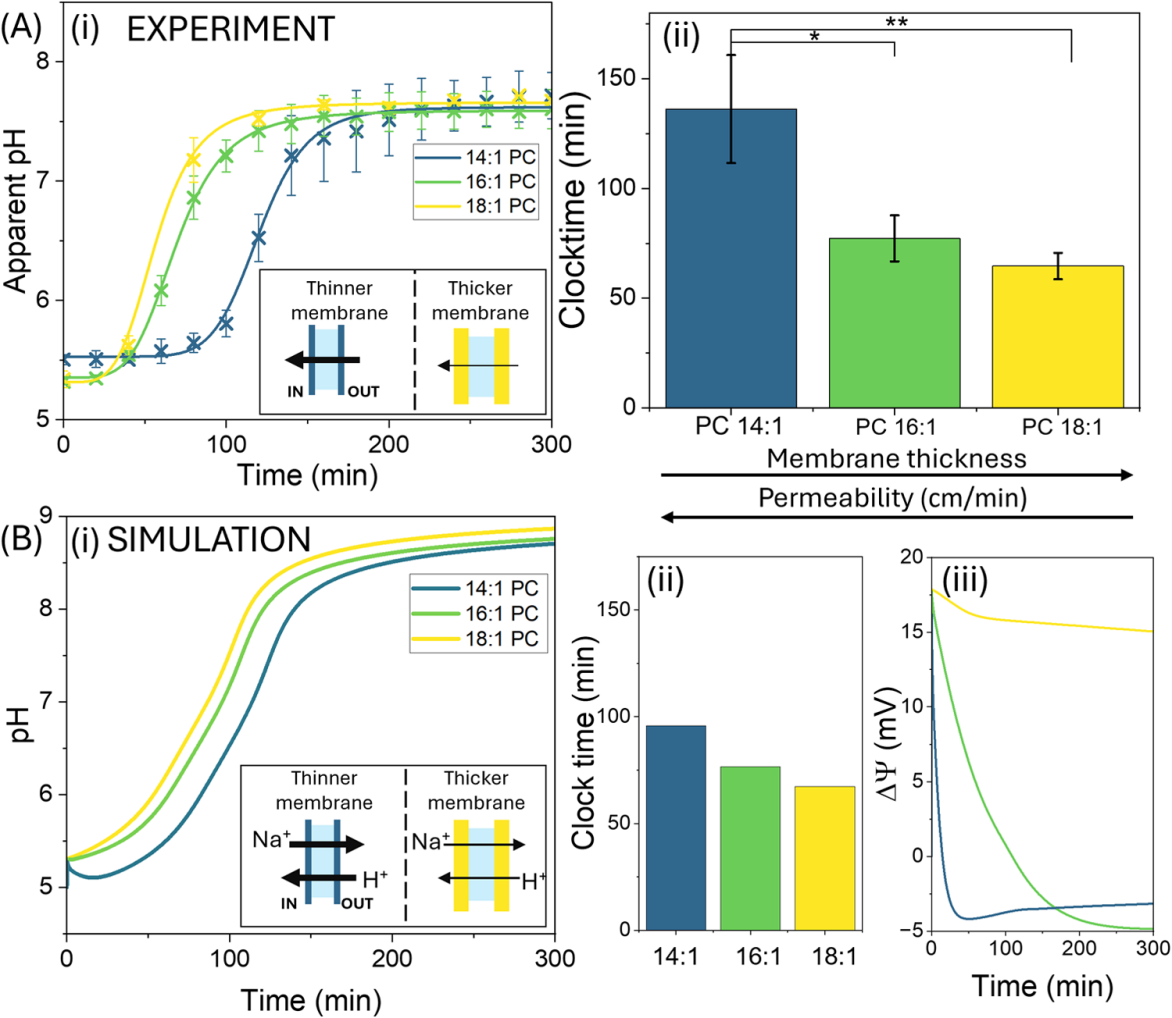
Enhanced confinement through increasing bilayer thickness speeds up the rate of the urease pH clock reaction inside vesicles. (A) The experimental change in pH over time in urease vesicles in acetate buffer with NaCl with different carbon chain lengths (18:1, 16:1, 14:1 PC) upon the addition of urea (*n* = 3, error = ± SE). (i) pH in time inside the vesicles. **Inset**: illustrations of increased permeability through thinner (blue) membrane compared to thicker (yellow) membrane. (ii) Experimental clock times of reactions shown in part (Ai) (*n* = 3, error = ± SE). (B) Simulations showing the effect of increasing permeability coefficients *P* on the pH clock in vesicles with increasing carbon chain length (*C* = 14 (blue), 16 (green) and 18 (yellow)). (i) pH in time inside the vesicles. (ii) Clock time as a function of chain length. (iii) Change in membrane potential in time. The *R*_max_ = 0.066 M/min and internal vesicle concentrations were: [H^+^] = 1 × 10^–5^ M, [acetate buffer] = 50 mM, [Na^+^] = 120 mM, [Cl^–^] = 70 mM and in the external solution: [urea] = 25 mM, [H^+^] = 1 × 10^–5^ M, [acetate buffer] = 25 mM; [Na^+^] = 50 mM, [Cl^–^] = 35 mM. The permeability coefficients were (cm/min): (*C* = 18) *P*_CO_2__ = 20, *P*_NH_3__ = 2, *P*_urea_ = 8.5 × 10^–5^, *P*_AA_ = 0.6, *P*_Cl–_ = 6 × 10^–7^, *P*_K+_ = 2 × 10^–10^, *P*_Na+_ = 2 × 10^–10^, *P*_H+_ = 1 × 10^–3^; (*C* = 16) *P* increased by a factor of 1.5 for neutral/anion and a factor of 100 for cations; (*C* = 14) *P* increased by a factor of 2 for neutral/anion and a factor of 800 for cations. For all other parameters see methods section.

Intuitively, we expected the reaction to run faster with a thinner membrane due to faster substrate transport into the vesicles where the enzyme is encapsulated. Surprisingly, the opposite trend was observed. As bilayer thickness decreased (18:1, 16:1, 14:1) the clock time in fact increased (65 ± 6, 77 ± 11, 140 ± 20 min, respectively in Figure 3Ai), (ii), indicating a slower reaction in a more permeable system.

This counterintuitive finding is clarified through the simulations of the system incorporating the dependence of permeability on membrane thickness for neutral species and ions Na^+^, Cl^–^ and H^+^. Transport of neutral species and small anions such as Cl^–^ typically follows a solubility diffusion mechanism, with permeability coefficients given by *P* = *KD*/*l* where *K* = solubility of the species in the hydrocarbon bilayer, *D* = diffusion coefficient and *l* = thickness of bilayer.^42^ Thus, permeabilities increased by a factor of 2 as bilayer thickness was decreased in monounsaturated PCs by decreasing the carbon chain length from *C* = 18 to 14.^40,47,50^ However, with cations H^+^ and K^+^ there was a dramatic increase in permeability as the phospholipid chain length was decreased below *C* = 18, suggesting that a different mechanism may become important, such as defect mediated transport.^50^ Anomalous high permeabilities of 1 × 10^–7^ cm/min were also obtained from experimental data with K^+^ and Na^+^ in POPC vesicles (16:0, 18:1 PC).^48,49^

Permeability values are not available for the membranes used in this work, however we increased them by a factor of 1.5 and 2 for neutral species/Cl^–^ and by a factor of 100 and 800 for cations with decreasing carbon chain length to *C* = 16 and 14 respectively, in line with the experimental trends.^50,51^ We observed that a thinner membrane allowed more sodium ion and proton flux out of and into the vesicle, respectively, maintaining a lower pH and increasing clock time, Figure 3Bi, (ii). For *C* = 18, the initial influx of protons as a result of the concentration gradient resulted in a high membrane potential, limiting further influx of protons under these conditions (Figure 3Biii). The fast counterflux of sodium ions was required to reduce the membrane potential, allowing more protons to enter thinner membranes. This reveals an unexpected but important effect of confinement on our reaction kinetics, highlighting how confinement and membrane dynamics are influential parameters in regulating feedback.

### Introducing Ion Transport

To further validate the predicted effects of membrane potential and proton transport in our experimental model, we imposed a K^+^ concentration gradient to counter the proton gradient and incorporated ionophores in the DOPC lipid membrane. This setup allows for additional proton movement into the vesicle lumen, countered by K^+^ moving in the opposite direction, in a near-electroneutral exchange. Ionophores are lipid-soluble compounds that can facilitate the transport of ions across lipid membranes,^52^ allowing us to explore the impact of ion movement and membrane potential on our pH-feedback system in vesicles with fixed membrane thickness.

To investigate the impact of proton transport, the protonophore carbonyl cyanide *m*-chlorophenyl hydrazone (CCCP) was introduced to catalyze the movement of protons down their concentration gradient. A potassium concentration gradient was generated across the membrane by encapsulating urease in acetate buffer with KCl inside the vesicles and NaCl in the buffer in the external medium, with equivalent 100 mM ionic strength. The ionophore valinomycin (Val) facilitates the transport of K^+^ ions down their concentration gradient with a specificity up to 100,000× greater than for Na^+^.^53^ The addition of 0.1 μM valinomycin to this system facilitates an outward movement of potassium ions, generating an inside negative electrochemical potential. The positive outside membrane potential will drive a slow leak of protons into the vesicle, which can be catalyzed further by the addition of CCCP.

**Figure 4:**
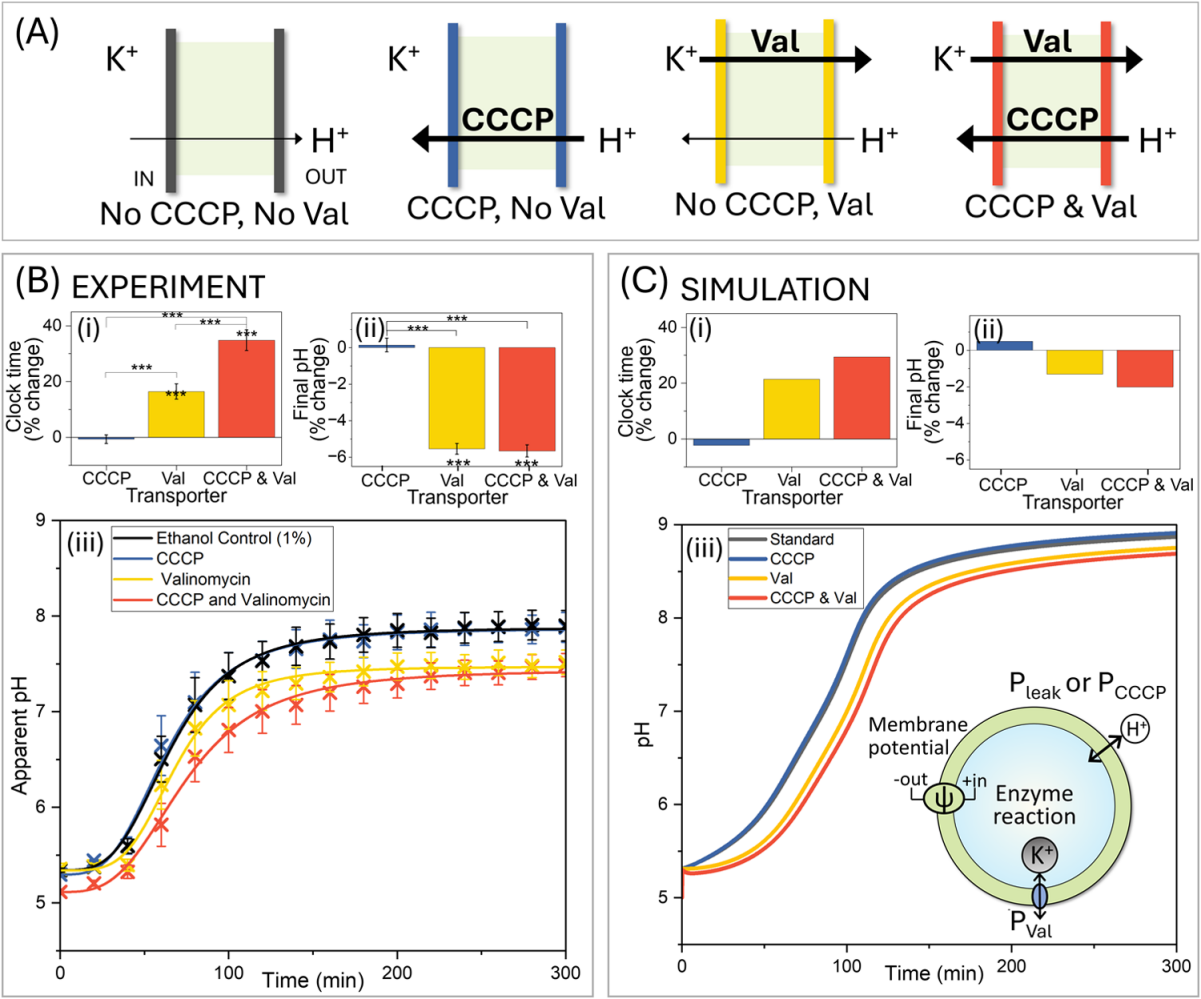
Modulating ion transport tunes the reaction kinetics of the encapsulated urea–urease clock reaction within lipid vesicles (A) Illustrations of different ion transport scenarios. (B) Experimental results for urease vesicles in acetate buffer loaded with KCl while external solution contained isomolar NaCl with 1% ethanol (black), 0.1 μM CCCP (blue), 0.1 μM valinomycin (yellow, Val) or 0.1 μM CCCP and 0.1 μM valinomycin (red, Val), upon the addition of urea solution. (i) Percentage change in clock time and (ii) final pH from 1% ethanol control (black) and (iii) the change in pH over time for all transporter conditions. (C) Simulated results for four conditions: standard case with negligible K^+^ permeability and small proton leak (black); increased proton permeability through CCCP addition (blue); valinomycin-only transport, allowing potassium permeability (yellow); and increased proton permeability and potassium permeability with the addition of both CCCP and valinomycin (red). (i) Percentage change in clock time and **(ii)** final pH from the standard case for all transporter scenarios, and **(iii)** pH time traces in vesicles. **Inset**: The cationic fluxes across the membrane in the model. This is controlled by a small proton membrane leak (*P*_leak_), CCCP facilitated proton flux (*P*_CCCP_), valinomycin-induced potassium permeability (*P*_Val_), and electrical potential across the membrane (ψ). The *R*_*max*_ = 0.066 M/min and internal vesicle concentrations were: [H^+^] = 1 × 10^–5^ M, [acetate buffer] = 50 mM, [K+] = 70 mM, [Na^+^] = 50 mM, [Cl^–^] = 70 mM and in the external solution: [urea] = 25 mM, [H^+^] = 1 × 10^–5^ M, [acetate buffer] = 25 mM; [K^+^] = 0 mM, [Na^+^] = 50 mM, [Cl^–^] = 35 mM. The permeability coefficients were (cm/min): *P*_CO_2__ = 20, *P*_NH_3__ = 2, *P*_urea_ = 8.5 × 10^–5^, *P*_AA_ = 0.6, *P*_Cl–_ = 6 × 10^–7^, *P*_K+_ = 2 × 10^–10^, *P*_Na+_ = 2 × 10^–10^, *P*_H+_ = 1 × 10^–3^. To simulate changes in permeability with CCCP and Val, a factor of 300 was used for *P*_H+_ and *P*_K+_. For all other parameters see Methods section.

In Figure 4, the clock reaction is presented for four distinct conditions: the 1% ethanol control with no facilitated ion transport (black); increased proton permeability through CCCP addition (blue); valinomycin-facilitated transport, allowing potassium permeability (yellow), and simultaneous increase in proton and potassium permeability with the addition of both CCCP and valinomycin (red). These conditions are illustrated in Figure 4A, with the experimental pH clock in Figure 4B and simulated pH clock depicted in Figure 4C.

Despite the initial internal pH being higher than the external pH, indicating a concentration gradient favoring proton influx into the vesicle core (Figure 4Ciii), addition of CCCP (0.1 μM) alone does not significantly affect clock time (black vs blue). This might appear surprising as we would expect increasing proton transport to influence a pH-dependent system. However, there are cases where increasing proton permeability, as explored with the model, has a limited effect on clock time due to the buildup of electrical potential limiting transport when there is a small capacitance. Consequently, only a few additional protons influx into the vesicle core, or protons may even leave the vesicle, depending on the magnitude of the opposing electrical gradient (see Figure 2D).

When the potential build-up due to proton influx is counterbalanced by K^+^ transport out of the vesicle via valinomycin (yellow), the final pH of the clock reaction is significantly reduced experimentally by 5.5% (1% for simulation) from the control (yellow vs black) and the clock time increases by 16.5% (21% in simulation). This is attributed to a slow leak of protons down the chemical gradient into the vesicles, acidifying the vesicle lumen and slowing the reaction rate.

The combined presence of CCCP and valinomycin (red) further enhances the movement of protons down the electrochemical gradient, increasing the acidification of the vesicle lumen and significantly increasing clock time by 35% (30% in simulation) from the control (red vs black) and lowering final pH by 5.7% (2% in simulation).

This has exposed how ion transport and membrane potential can govern the reaction dynamics of our pH-feedback enzyme system, with convincing correspondence between the trends in the experimental and numerical data. Additionally, we find that under the current conditions, the effect of enhancing proton permeability (blue) on our enzyme reaction is largely restricted by the electrical gradient, only becoming an influential parameter once thermodynamic conditions are met, demonstrating why membrane potential needs careful consideration.

### Choice of Buffer

**Figure 5:**
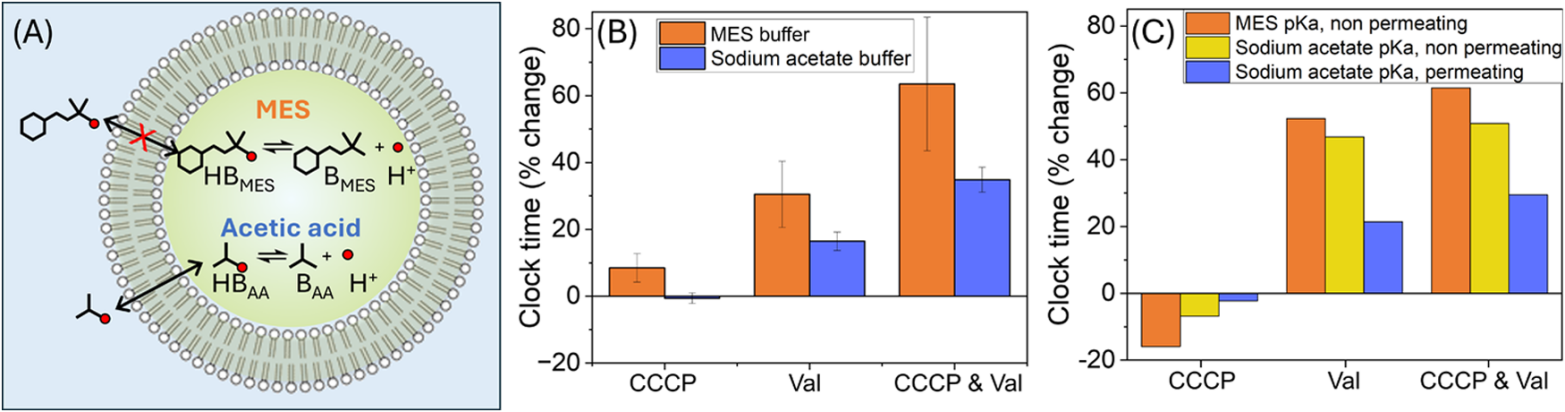
Choice of buffer enhances the effects of proton transport driven by an imposed membrane potential. (A) Schematic of acetic acid (permeating, p*K*_a_ 4.76) and MES (nonpermeating, p*K*_a_ 6.15) buffers. (B) Percentage change in clock time (time to reach pH = 6.4) from 1% ethanol control for all transporter conditions of urease vesicles in MES buffer or sodium acetate buffer loaded with KCl while external solution contained NaCl (*n* = 3, error = ± SE) and mixed with urea solution. (C) Percentage change in simulated clock time for all transporter conditions performed in sodium acetate buffer (blue, p*K*_a_ 4.76, permeable) and MES buffer (orange, p*K*_a_ 6.15, nonpermeable), compared with nonpermeable acetate buffer (yellow, *P*_AA_ = 0). Simulations were performed with *R*_*max*_ = 0.066 M/min and internal vesicle concentrations were: [H^+^] = 1 × 10^–5^ M, [acetate or MES buffer] = 50 mM, [K^+^] = 70 mM, [Na^+^] = 50 mM, [Cl^–^] = 70 mM and in the external solution: [urea] = 25 mM, [H^+^] = 1 × 10^–5^ M, [acetate or MES buffer] = 25 mM; [K^+^] = 0 mM, [Na^+^] = 50 mM, [Cl^–^] = 35 mM. The permeability coefficients were (cm/min): *P*_CO_2__ = 20, *P*_NH_3__ = 2, *P*_urea_ = 8.5 × 10^–5^, *P*_AA_ = 0.6, *P*_Cl–_ = 6 × 10^–7^, *P*_K+_ = 2 × 10^–10^, *P*_Na+_ = 2 × 10^–10^, *P*_H+_ = 1 × 10^–3^. To simulate changes in permeability with CCCP and Val, an increase in permeability by a factor of 300 was used for *P*_H+_ and *P*_K+_. For all other parameters see methods section.

Both acetic acid (from the acetate buffer) and ammonia can contribute to the dissipation of the proton gradient.^52^ Acetic acid is a weak acid and, in its un-ionized form, serves as a neutral proton carrier, facilitating transmembrane H^+^ translocation without inducing a transmembrane electrostatic potential (Figure 5), and therefore diminishes the effects of proton carriers as seen in the previous experiment (Figure 4).^52^ To further elucidate this point, we conducted parallel experiments using an isomolar pH 5 MES buffer, which has a much lower ability to permeate the membrane. In comparison to experiments using the sodium acetate buffer, reactions performed in MES buffer exhibited greater variability, and the extrusion of vesicle samples was notably more challenging, suggesting these buffer molecules were less favorable for the urease enzyme. However, across all conditions (CCCP, Val, CCCP and Val), the increase in clock time compared to the standard condition is more pronounced with MES buffer than with sodium acetate (Figure 5B).

This trend is consistent with our theoretical model upon replacing acetic acid buffer with MES, which shows that the clock time depends on both the high p*K*_a_ of MES and the absence of proton movement through acetic acid (Figure 5C). The clock time increased significantly as a result of the influx of H^+^ with Val, or CCCP and Val, if the permeability of acetic acid was set to zero (yellow), and further increased when the p*K*_a_ was raised to that of MES, 6.15 (orange). These experiments demonstrate that the absence of proton permeation through the buffer significantly enhances the reliance on CCCP-mediated proton transport and the role of valinomycin in proton decoupling. It underscores the importance of selecting the appropriate buffer in lipid vesicle systems, not only with considerations of its p*K*_a_ but also its ability to permeate the membrane and equilibrate pH.

## Overview and Conclusions

### Implications of Confinement and Electrochemical Gradients on Enzyme Activity

The influence of lipid vesicles on enzyme activity is well-established, particularly regarding the creation of microenvironments that affect substrate availability and enzyme kinetics.^54^ However, few studies have demonstrated enhanced catalytic activity due to increased confinement. Luisi et al. encapsulated a cell-free expression system, comprised of 83 components, in 100 nm lipid vesicles and demonstrated it was capable of fluorescent protein synthesis. Despite the low probability of all 83 components being encapsulated together, which statistically should have resulted in only a negligible fraction of viable vesicles, the expression of fluorescent protein in the lipid vesicles was six times higher than in bulk water. This was hypothesized to result from a concentration enrichment effect due to an expulsion of water from the lipid vesicles.^55^

Other enzyme studies (chymotrypsin, HRP, lipase), predominantly performed in reverse micelles, have reported a “superactivity” effect. This enhancement is proposed to be due to structural changes, proximity effects of enzyme and substrate, or due to the physicochemical dynamics of water.^3,56,57^ Similarly, catalytic enhancement has also been observed using DNA scaffolds for enzyme assembly and immobilization, inspired by scaffolds in cells to colocalize enzyme and substrate. The negatively charged surface of DNA is interpreted to lead to catalytic enhancements by increasing the substrate’s affinity via electrostatic forces, lowering local pH, and providing an organized hydration layer.^58−61^ Our study contributes to the understanding of confinement-enhanced enzyme catalysis by introducing another potential mechanism: where the lower permeability of a thicker membrane could enhance catalysis through increased protection from protons in the external solution.

The broader implications of electrochemical gradients on intracellular pH, especially in artificial cells, have been largely overlooked. Subtle changes in intracellular pH can significantly impact cellular activity and physiology. For example, the minor 0.2 unit differences in lysosomal pH due to a mutation in a Cl^–^/H^+^ exchanger have been associated with developmental delay and hyperpigmentation.^62^ In our study, we uncover how membrane potential can modulate enzyme activity through pH feedback within our reactive lipid vesicles with a significantly large increase of 35% in clock time from the control in the presence of valinomycin and CCCP. Additionally, in our minimal system we find proton permeability and electrical gradient are implicitly interlinked, finding certain scenarios where proton transport is not significantly limited by a kinetic barrier, but rather by whether the thermodynamic requirements are met. In bioenergetic systems, proton permeability is the dominating mechanism as proton pumping establishes the electrical gradient.^7^ However, in artificial cells systems, if the electrical gradient is formed by mechanisms other than proton pumping, permeability may not dominate and the membrane potential should be carefully considered.

These significant findings in a minimal cell system offer insights into how membrane potential and counterion regulatory mechanisms could govern cellular homeostasis and bioreactions, underscoring the need to consider these interactions for a comprehensive understanding of cellular physiology. Furthermore, our findings have shown it is imperative to consider electrochemical gradients and intracellular pH to understand and control the functionality of artificial cells, especially in a context where final pH and enzyme reaction rates are important.

## Conclusions

In this work, we have developed a simplified model, both experimentally and numerically, to explore how membrane transport and confinement affect pH feedback mechanisms of enzyme reactions within lipid vesicles, revealing complex and significant interactions. Despite conventional expectations that thinner bilayers would enhance substrate permeability and enzyme reaction rates,^3^ we observed that a thinner membrane actually prolonged reaction times. This counterintuitive phenomenon is explained by the reduced membrane permeability of protons with thicker membranes, augmenting the localized base-catalyzed feedback mechanism inside the vesicles. Additionally, we have elucidated how membrane potential and ion transport can modulate pH-dependent enzymatic activity with implications in artificial cell and bioinspired vesicle design. This is demonstrated through an ion transport induced membrane potential that drives the influx of protons into vesicles, maintaining a low pH environment for longer and slowing the rate of the internal pH clock reaction. This effect is further amplified by suppressing parallel pathways for proton leakage across the membrane, in this case by changing the buffer. These findings offer valuable insights into cell chemical organization and offer knowledge crucial for designing synthetic cells with specific controlled functionalities. Future research could further explore the interplay between membrane dynamics of individual vesicles, delving further into their collective behavior. Overall, our findings will be crucial for designing artificial cells or organelles for biomedical applications or other technologies where maintaining or controlling the specific internal pH is important.

## Methods

### Construction of Urease Vesicles

The thin film hydration and extrusion method was used to make 1,2-dioleoyl-*sn*-glycero-3-phosphocholine (DOPC) vesicles (17 mM lipid) encapsulating pyranine (50 μM) and urease (20 μM; 11 mg/mL of type III, Sigma-Aldrich) in 50 mM sodium acetate buffer (100 mM ionic strength adjust with NaCl) at pH 5, extruded through 0.2 μm filter (see SI 1–4 for more details). Unencapsulated urease and pyranine were removed using Superose 6 Increase 10/300 GL (Cytiva) connected to an ÄKTA chromatography system (Cytivia). Urease vesicles (2.8 ± 0.4 mM DOPC from phosphorus assay (SI 1.3.3.) with hydrodynamic diameter of 164 ± 3 nm from DLS (SI 1.2.4)) in 50 mM sodium acetate buffer (100 mM ionic strength adjusted with NaCl) was collected and reactions were initiated with 50 mM urea at a 1:1 volume ratio. Change in fluorescence intensity ratio (excitation 450/405 nm) of pyranine was measured using a FluoroMax-3 fluorometer, with emission at 511 nm every 10 min and converted to apparent pH using a calibration curve (SI 1.2.1). Purification of urease vesicles was confirmed using pyranine vesicles, where 20 μM urease was added externally followed by size exclusion chromatography. A reaction with collected pyranine vesicles and any remaining external urease was initiated using 50 mM urea at a 1:1 volume ratio, where pH change was monitored using the fluorescence intensity ratio (450/405 nm) of pyranine. As no pH change was found, vesicles had been sufficiently purified from external urease.

### Changing Bilayer Thickness

20 μM urease (11 g/mL of type III, Sigma-Aldrich) and 50 μM pyranine were encapsulated in lipid vesicles (164 ± 3 nm diameter from DLS (SI 1.2.4)) using thin film hydration and extrusion with either DOPC (18:1 (Δ9-Cis) PC), 1,2-dipalmitoleoyl-*sn*-glycero-3-phosphocholine (16:1 (Δ9-Cis) PC) or 1,2-dimyristoleoyl-*sn*-glycero-3-phosphocholine (14:1 (Δ9-Cis) PC) lipids (17 mM) in 50 mM sodium acetate buffer (pH 5, 100 mM ionic strength adjust with NaCl). After undergoing size exclusion chromatography, 100 μL of vesicles were placed into plate wells before 100 μL of urea (50 mM) was added to initiate the reaction. Change in fluorescent intensity ratio (450/405 nm) of pyranine was measured using the EnVision 2105 multimode plate reader and converted to apparent pH (SI 1.2.1).

### Introducing Ion Transport

20 μM urease (11 g/mL, type III Sigma-Aldrich) and 50 μM pyranine vesicles were made up in sodium acetate buffer (50 mM, pH 5, 100 mM ionic strength KCl). Size exclusion chromatography was used to remove unencapsulated species and exchange buffer to sodium acetate buffer (50 mM, pH 5, 100 mM ionic strength NaCl). 99 μL of vesicles was added to 4 wells before 1 μL of CCCP, valinomycin or CCCP and valinomycin solubilized in ethanol were added to 3 wells to a concentration of 0.2 μM. 1.0 μL of ethanol was added to the control so all 4 samples are 1% ethanol. A reaction was initiated through the addition of 100 μL of urea (50 mM, pH 5) to give a final concentration of 25 mM. Change in fluorescent intensity ratio (450/405 nm) of pyranine was measured using the EnVision 2105 multimode plate reader and converted to apparent pH (S1 1.2.1.)

### Removing Intrinsic Proton Transport by Acetic Acid

The same procedure as above (Introducing ion transport) was used except 50 mM MES buffer (pH 5, 100 mM ionic strength KCl) was used for the assembly of lipid vesicles (containing urease and pyranine at the previously noted concentrations). During size exclusion chromatography, the running buffer was 50 mM MES buffer (pH 5, 100 mM ionic strength NaCl).

### Simulations

The system was simulated using reactions (1) – (7) shown in Figure 1B resulting in a 35-variable ODE model describing the rates of change of species in vesicles and external solution. The rate constants for the urease reaction were taken from previous work.^31^ The model incorporates urease-catalyzed urea hydrolysis from a modified Michaelis–Menten equation with Michaelis constant *K*_M_ = 0.003 M; inhibition constants *K*_S_ = 3 and *K*_p_ = 0.2; acid binding constants *K*_es1_ = 5 × 10^–6^ and *K*_es2_ = 2 × 10^–9^ and maximum rate *R*_max_ = *k*_1_[*E*]_0_ where *k*_1_ = turnover number (∼10^3^ - 10^4^ s^–1^) and [*E*]_0_ = total enzyme concentration. The pH equilibria follow mass action kinetics with *k*_2_ = 1440 min^–1^; *k*_2r_ = 2.58 × 10^12^ M^–1^ min^–1^; *k*_3_ = 2.22 min^–1^; *k*_3r_ = 4.74 × 10^6^ M^–1^ min^–1^; *k*_4_ = 168 min^–1^; *k*_4r_ = 3 × 10^12^ M^–1^ min^–1^; *k*_5_ = 6 × 10^–2^ M min^–1^; *k*_5r_ = 6 × 10^12^ M^–1^ min^–1^; *k*_6_ = 60 min^–1^; *k*_6r_ = 1.5 × 10^9^ M^–1^ min^–1^; *k*_7r_ = 2.7 × 10^12^ M^–1^ min^–1^; *k*_7_ = 4.68 × 10^7^ min^–1^ (acetic acid); *k*_7_ = 1.9 × 10^6^ min^–1^ (MES), *k*_7r_ = 2 × 10^12^ M^–1^ min^–1^.

For the standard conditions of the DOPC (*C* = 18) vesicle experiment (Figure 3A), *R*_max_ = 0.066 M/min. The permeability coefficients of species were taken as (cm/min): *P*_CO2_ = 20, *P*_NH3_ = 2, *P*_urea_ = 8.5 × 10^–5^, *P*_AA_ = 0.6, *P*_Cl–_ = 6 × 10^–7^, *P*_K+_ = 2 × 10^–10^, *P*_Na+_ = 2 × 10^–10^, *P*_H+_ = 1 × 10^–3^ in line with literature,^40,41,47−50^ and the membrane capacitance as *C*_0_ = 0.8 μF/cm^2^,^44,45^ All other ion permeability coefficients were set to zero. The vesicle volume *V* and surface area *S* were calculated from the vesicle diameter, taken as 160 nm, assuming a spherical shape. The vesicle volume fraction was fixed throughout as ϕ = 2 × 10^–3^.

The initial concentrations, unless otherwise stated, were taken from the experimental conditions: in the vesicles, [pyranine] = 50 μm, [H^+^] = 1 × 10^–5^ M, [OH^–^] = 1 × 10^–9^ M, [acetate buffer] = 50 mM ([acetate] = 32 mM, [acetic acid] = 18 mM), [Na^+^] = 120 mM (from NaCl, sodium acetate and NaH_2_PO_4_ in type III enzyme powder), [Cl^–^] = 70 mM (from NaCl) and in the external solution: [urea] = 25 mM, [H^+^] = 1 × 10^–5^ M, [acetate buffer] = 25 mM ([acetate] = 16 mM, [acetic acid] = 9 mM); [Na^+^] = 50 mM (from NaCl and sodium acetate), [Cl^–^] = 35 mM (from NaCl). All other initial concentrations were set to zero. The equations were solved using MATLAB and stiff solver odes15. Full details of the model, including all assumptions, can be found in SI 3.

## Supporting Information Available

Materials and extended methods; preparation of lipid vesicles with encapsulated urease; calibration of pyranine for apparent pH; lipid vesicle synthesis and characterization; urease lipid vesicle encapsulation efficiency; lipid vesicle concentration; and effect of valinomycin and CCCP on urease bulk clock time. Also, there is the modeling of the urea–urease reaction in lipid vesicles: urease-catalyzed reaction; pH equilibria; transfer rates and permeability; model equations and parameters; and model assumptions (PDF)
