# Supplementary material for: Membrane Transport Modulates the pH-Regulated Feedback of an Enzyme Reaction Confined within Lipid Vesicles
Source: ACS Nano. 2025 Mar 3;19(10):9814–25. doi: 10.1021/acsnano.4c13048 (PMC11924318; doi:10.1021/acsnano.4c13048)
Supplement: Supplementary file 1 — nn4c13048_si_001.pdf [file nn4c13048_si_001.pdf]

# Supporting Information

## Membrane transport modulates the pH-regulated feedback of an enzyme reaction confined within lipid vesicles

Darcey Ridgway-Brown‡,[1,2] Anna S. Leathard‡,[3] Oliver France,[1,2] Stephen P. Muench,[2,4] Michael E. Webb,[1,2] Lars J. C. Jeuken,[5] Peter J. F. Henderson,[2,4] Annette F. Taylor,[3,6,*] Paul A. Beales.[1,2,*]

1. School of Chemistry, University of Leeds, Leeds LS2 9JT, U.K
2. Astbury Centre for Structural Molecular Biology, University of Leeds, Leeds LS2 9JT, U.K.
3. Chemical and Biological Engineering, University of Sheffield, Sheffield S1 3JD, U.K.
4. School of Biomedical Sciences, Faculty of Biological Sciences, University of Leeds, Leeds LS2 9JT, U.K.
5. Leiden Institute of Chemistry, University Leiden, PO Box 9502, 2300 RA Leiden, The Netherlands.
6. School of Chemistry and Chemical Engineering, University of Southampton, Southampton SO17 1BJ, U.K.

* Corresponding authors: AFT (a.f.taylor@soton.ac.uk); PAB (p.a.beales@leeds.ac.uk)

# Contents

Contents ........ 2
1. Materials and extended experimental methods ........ 3
1.1. Materials ........ 3
1.2. Preparation of lipid vesicles with encapsulated urease ........ 3
1.2.1. Calibration of pyranine for apparent pH ........ 3
1.2.2. Extracting clock time ........ 4
1.2.3. Lipid vesicle synthesis ........ 4
1.2.4. Dynamic light scattering (DLS) ........ 5
1.3. Urease Activity Assay and Encapsulation Efficiency ........ 5
1.3.1. Urease activity assays ........ 5
1.3.2. Encapsulation efficiency assay ........ 6
1.3.3. Phosphorous assay ........ 7
2. Valinomycin and CCCP- effect on urea-urease reaction in bulk ........ 8
3. Modelling of the urea-urease reaction in liposomes ........ 9
3.1. Urease-catalysed reaction ........ 9
3.2. pH equilibria ........ 9
3.3. Transfer rates and permeability ........ 10
3.3.1. The transfer of neutral species ........ 10
3.3.2. The transfer of ionic species ........ 10
3.3.4. Variation according to membrane length ........ 11
3.4. Model equations and parameters ........ 12
3.5. Model assumptions ........ 13
References ........ 14

# 1. Materials and extended experimental methods

## 1.1. Materials

The pH indicator 8-hydroxypyrene-1,3,6-trisulfonic acid trisodium salt (pyranine) and Jack bean urease (JBU) from *Canavalia ensiformis* (type III) were purchased from Sigma-Aldrich. All lipids were purchased from Avanti Polar lipids Inc. Valinomycin and carbonyl cyanide 3-chlorophenylhydrazone (CCCP) were purchased from Sigma-Aldrich. Note, JBU is the only urease investigated and will be referred to as urease.

For the urease type III used in the experiments, the typical specific activity was ~30 units/ mg where 1 unit (U) = 1 µmol $NH_3$ $min^{-1}$ at pH 7 and 25 ˚C. The molecular mass of urease is $M_r$ = 545 kDa. A concentration of 20 µM urease corresponds to 11 mg/ mL and activity ~330 U/ mL assuming pure enzyme. However, type III contains impurities including phosphates from the purification process; hence 20 µM is an upper limit of the concentration. Purified urease (type C3) has reported specific activities of >600 U/ mg, hence we estimate <50% of the mass is phosphate in a prepared urease sample. Assuming 30% sodium dihydrogen phosphate, this gives a concentration of $[Na^+]$ ~ 20 mM.

## 1.2. Preparation of lipid vesicles with encapsulated urease

### 1.2.1. Calibration of pyranine for apparent pH

Pyranine is a ratiometric fluorescent pH indicator that can be used to monitor pH inside vesicles. Pyranine is ratiometric due to its two possible pH-dependent excitation maxima resulting from its different protonation states; 405 nm when protonated and 450 nm when deprotonated. Therefore, a fluorescent intensity ratio (450/405 nm) can be calculated, mitigating for variability in concentrations, which would lead to fluctuations in fluorescent intensity.

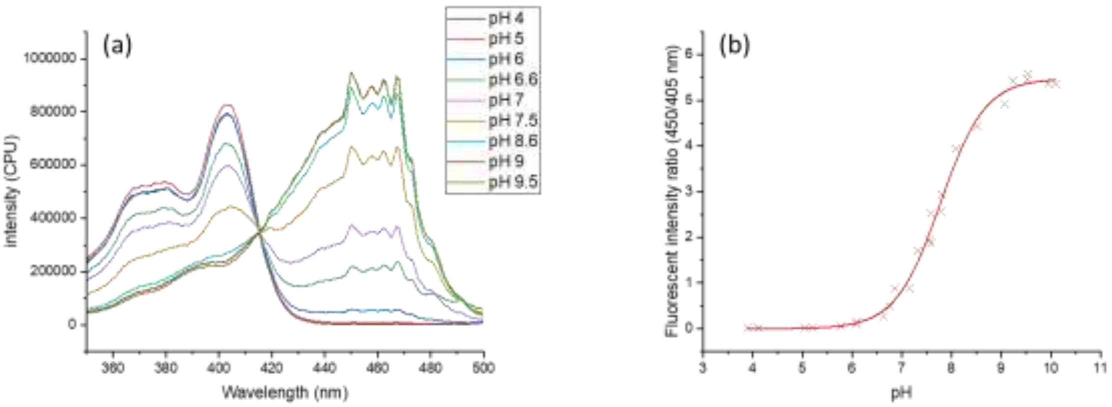


**Figure S1. Fluorescent intensity of pyranine (0.25 µM) at a range of pH values in sodium acetate buffer.** (a) Excitation spectrums of pyranine on the fluorometer at different pH values measured at 511 nm, showing how the pyranine fluorescence profile shifts due to change in protonation state. (b) Calibration curve of pyranine fluorescence intensity ratio (450/405 nm) against pH on a plate reader, with fitted equation (line): $y = a + (b - a)/(1 + 10d*(c-x))$ where a = -0.003 ± 0.078, b = 5.5 ± 0.1, c = 7.78 ± 0.03 and d = 0.96 ± 0.08.

A calibration curve of the fluorescent intensity ratio (450/405 nm) of 8-hydroxypyrene-1,3,6-trisulfonic acid trisodium salt (pyranine) (0.25 µM) at a range of pH values was obtained in 50 mM sodium acetate buffer (100 mM ionic strength, adjusted with NaCl). At each pH, an excitation spectrum from 350 nm to 500 nm was measured using a FluoroMax-3 fluorometer, with emission at 511 nm. Fluorescent intensity at 450 nm and 405 nm was used to calculate a fluorescent intensity ratio (450/405 nm) of pyranine, which was plotted against pH. Change in fluorescent intensity ratio (450/405 nm) of pyranine was also measured using the EnVision® 2105 multimode plate reader to create a second calibration curve. To closely match *in situ* fluorescent intensity values of pyranine within vesicles, calibration curves were created in the presence of liposomes.

$$\mathrm{PyOH^{3-}} \rightarrow \mathrm{PyO^{4-}} + \mathrm{H^{+}} \qquad K_a = \frac{[PyO^{4-}][H^{+}]}{PyOH^{3-}} \tag{SE1}$$

$$R = R_{min} + \frac{R_{max} - R_{min}}{1 + 10^{(pKa' - \mathrm{pH})}} \tag{SE2}$$

$$\mathrm{pH} = \mathrm{p}K_a{}' - \log\left(\frac{R - R_{max}}{R_{min} - R}\right) \tag{SE3}$$

Calibration data was fitted using $y = a + (b - a)/(1 + 10^{d*(c - x)})$ (SE2) in OriginPro. The apparent pH was determined from SE3: $\mathrm{pH} = c - (1/d)\log((y - b)/(a - y))$ and the error in the pH, $\partial$pH, was related to the error in R ($\partial y$) using the propagation of error formula: $\partial\mathrm{pH} = (\partial y^2((y - b)/(y - a)^2 - 1/(y - a))^2(y - a)^2)/(d^2(y - b)^2))^{1/2}/\ln(10)$.

### 1.2.2. Extracting clock time

All data was analysed using OriginPro. All clock reactions were fitted with Hill1 with equation $y = \mathrm{START} + (y\_f - y\_0)*(x^n/(k^n+x^n))$. We determined the average pH midpoint of acid-base switching behaviour by calculating the mean between the start and end pH value of all clock reactions (pH 6.4). From the fitted relationship, clock time was defined as the time to reach pH 6.4. Extracted clock time values were analysed using one-way repeated ANOVA, followed by Tukey's multiple comparisons test, where a p-value > 0.05 was considered not significant.

### 1.2.3. Lipid vesicle synthesis

A volume of 1 ml of 17 mM lipid in chloroform was placed in a glass vial. The chloroform was evaporated using nitrogen gas, developing a dry lipid film. The vial was then dried overnight under vacuum to remove remaining solvent. The desiccated lipid film was rehydrated in 1 ml of relevant encapsulant solution (e.g. urease (20 µM) and pyranine (50 µM) in 50 mM sodium acetate buffer (100 mM ionic strength with NaCl) and vortexed to create a 17 mM lipid suspension. The solution was freeze-thawed ten times in liquid nitrogen, to homogenize the suspension and decrease vesicle lamellarity before extruding eleven times through a 200 nm pore polycarbonate membrane (Whatman, Ltd) using a mini-extruder (Avestin, Inc). This produced 164 ± 3 nm diameter large unilamellar vesicles (LUVs) with a PdI of 0.13, confirmed using Dynamic Light Scattering (DLS). Unencapsulated enzyme

and pyranine were removed using a Superose® 6 Increase 10/300 GL (Cytiva) connected to an ÄKTA chromatography system (Cytivia) and eluted in the appropriate buffer, as described in main methods.

### 1.2.4. Dynamic light scattering (DLS)

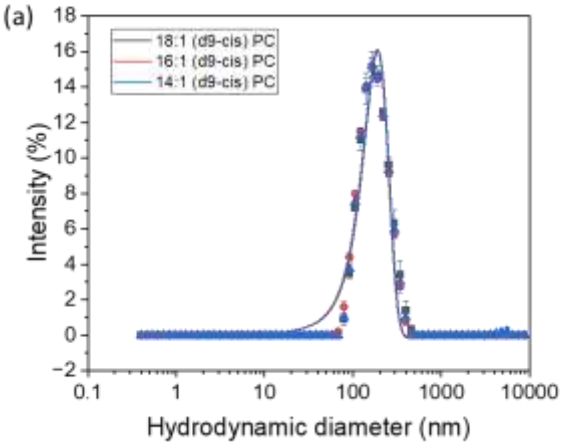


(b)

| Lipid chain length | PdI | Z-Ave (d.nm) |
|---|---|---|
| 18:1 (d9-cis) PC | 0.14 | 167 |
| 16:1 (d9-cis) PC | 0.13 | 164 |
| 14:1 (d9-cis) PC | 0.11 | 159 |

**Figure S2.** (a) Dynamic light scattering (DLS) intensity profiles of lipid vesicles with different lipid chain lengths (b) DLS data reporting Polydispersity Index (PdI) and Z-average hydrodynamic diameter (Z-Ave in nm) of lipid vesicles with different lipid chain lengths.

Backscatter of photons by particles in the sample was measured at a 173° angle at 25 °C after a 120 second equilibration using a ZetaSizer Nano ZSP and interpreted by ZetaSizer software (Malvern Instruments) to measure the intensity auto-correlation function, analysed using second order cumulant method.

## 1.3. Urease Activity Assay and Encapsulation Efficiency

### 1.3.1. Urease activity assays

Urease activity was determined by adapting the well-established Berthelot reaction to measure ammonia production from urea hydrolysis over time.[1] In triplicate, an aliquot of sample containing urease was incubated at 37 °C in 150 mM phosphate buffer at pH 7 with 50 mM urea for a total volume of 1 ml for 10 min. During this time, the two Berthelot reagents were mixed at a 1:1 ratio. Reagent 1 contains 0.17 M sodium salicylate, 3 mM sodium nitroprusside and 0.5 M NaOH. Reagent 2 contains 0.03 mg/ml sodium hypochlorite (10-15% available Chlorine) and 1 M NaOH. After 10 min, each reaction was quenched through the 1 ml addition of 1:1 Berthelot reagents. The samples were covered from light and incubated at 45°C for 30 min in the dark, allowing the colour to develop. A calibration curve of known ammonia concentrations was created using ammonium chloride in 150 mM phosphate buffer each time. 50 mM urea was added at 37 °C for 10 min to each calibration point before the 1 ml addition of 1:1 Berthelot reagents to account for spontaneous hydrolysis of ammonia before incubation at 45°C for 30 min in the dark. After 30 min, absorbance at 660 nm was measured using a Cary 100 ultraviolet-visible spectrometer. A calibration curve of absorbance of known ammonia concentration was created to determine unknown ammonia concentrations of urease samples. From this, ammonia production over

10 min was determined and therefore urease activity of sample (u/ ml) can be calculated, taking dilution factors into account, where 1 u is defined as the amount of enzyme required to convert 1 µmol $NH_3$ $min^{-1}$ at pH 7 at 37°C.

### 1.3.2. Encapsulation efficiency assay

Following thin-film hydration and extrusion, a 5 µl aliquot of vesicle sample was taken to serve as a 'total urease' sample. The lipid vesicles were then loaded onto a Superose® 6 Increase 10/300 GL (Cytiva) connected to an ÄKTA chromatography system (Cytivia) to remove external urease. The volume loaded was noted and a 3 ml fraction of lipid vesicles was collected. The 5 µl 'total urease' sample was diluted to 1 ml in 150 mM phosphate buffer at pH 7 with 0.3% Triton X-100. A 180 µl aliquot of urease-encapsulated vesicles was added to 90 µl 150 mM phosphate buffer at pH 7 with 0.3% Triton X-100. In triplicate, 20 µl aliquots of both diluted samples were incubated at 37 °C in 150 mM phosphate buffer at pH 7 and 50 mM urea for a total volume of 1 ml for 10 min, following the procedure in section 1.3.1 (urease activity assay). The urease activity (u/ ml) of both samples was determined.

The total volume of liposomal lumen in the original 1 ml sample was calculated based on the lipid concentration. This calculation finds the theoretical number of lipids in each vesicle (SE4) based on the average vesicle size measured by DLS. The number density of liposomes in the sample can then be calculated based on the lipid concentration determined by a phosphorus assay. Typical values where $d$ = liposome diameter (164 nm from DLS), $h$ = thickness of bilayer (5 nm), $a$ = lipid head group area (0.71 $nm^2$), $M_{lip}$= molar concentration of lipids (17 mM) and $N_A$= Avogadro's number (6.02 × $10^{23}$) were used to calculate $N_{lip}$, the number of lipids in each liposome (244619) and $N_{ves}$, the number of vesicles in the sample (4.57 × $10^{13}$).[2]

$$N_{lip} = \frac{4\pi\left(\frac{d}{2}\right)^2 + 4\pi\left(\frac{d}{2}-h\right)^2}{a} \qquad \text{(SE4)}$$

$$N_{ves} = \frac{M_{lip} \times \mathrm{N}_A}{N_{lip} \times 1000} \qquad \text{(SE5)}$$

The theoretical volume of one liposomal lumen ($V_i$) was calculated (SE6) as 1.07 ×$10^{-15}$ ml from which the total volume of liposomal lumen in original 1 ml sample ($N_{ves}$ x $V$i) compared to the outer 1 ml volume, known as the volume fraction (SE7) was determined. This was calculated to be 0.048.

$$V_i = \frac{3}{4}\pi \times \left(\frac{d}{2} - h\right)^3 \qquad \text{(SE6)}$$

$$\Phi' = \frac{N_{ves}\, V_i}{V_o} \qquad \text{(SE7)}$$

While known dilution factors are taken into account during the activity assay, there is an unknown loss of vesicles to the column due to the retention of liposomes.[3] Using a phosphorous assay (SI 1.3.3.), the phosphorous content and so lipid concentration of vesicles samples was calculated before and after size exclusion chromatography to determine vesicle sample loss. From this, a dilution factor of 1.7 ± 0.3 due to purification was calculated. This is in addition to the 1:3 dilution due to 1 ml sample being loaded and a 3 ml fraction being collected during size exclusion chromatography, and an additional 1:1 volume addition of urea (50 mM) during a reaction, which leads to a final volume fraction of the reactive vesicles (SE8) of 0.0047; i.e. of the order of $10^{-3}$, taking into account the assumptions (uniform size, no loss of vesicles upon addition of urea).

$$\Phi = \frac{\Phi'}{1.7 \times 3 \times 2} \qquad \text{(SE8)}$$

### 1.3.3. Phosphorous assay

To calculate the loss of vesicles that occurs during the preparation process of urease vesicles, a phosphorous assay can be used to determine the phosphorous content of lipid samples before and after size exclusion chromatography. Five phosphorous standards and a blank were placed into six borosilicate test tubes: 0 µmoles (0 µl) blank, 0.0325 µmoles (50 µl), 0.065 µmoles (100 µl), 0.114 µmoles (175 µl), 0.163 µmoles (250 µl), and 0.228 µmoles (350 µl). All were made up to 350 µl with deionised water. In triplicate, test samples were added to additional test tubes and made up to 350 µl.

A volume of 0.45 ml of 8.9 N $H_2SO_4$ was added to each test tube before heating at 210 °C for 25 min. The samples were then cooled for 5 min before adding 150 µl 30% w/w $H_2O_2$ to the bottom of all tubes. The test tubes were heated for a further 30 min at 210 °C. The test tubes were then removed and cooled for 5 min, before 3.9 mL deionised water, 0.5 ml of 2.5% w/v ammonium molybdate (VI) tetrahydrate solution, and 0.5 ml of 10% w/v ascorbic acid solution were added, vortexing between additions. Each tube was capped before heating for 7 min at 100°C. The test tubes were then cooled to room temperature. The absorbance of each calibration standard and test sample were measured at 820 nm using a Cary 100 UV-vis spectrometer. A calibration curve was generated from the standards and used to determine the phosphorous concentration of the vesicle samples.

# 2. Valinomycin and CCCP- effect on urea-urease reaction in bulk

To be confident the increased clock time is due to changes in permeability, we must establish whether CCCP or valinomycin effects the reaction in bulk conditions, to ensure the increased clock time is not due to an interaction with any other species. A solution of urease (3 µM) and pyranine (0.5 µM) was made in 50 mM sodium acetate buffer (pH 5, 100 mM ionic strength with NaCl) with 1 % ethanol containing either CCCP, valinomycin, both or neither at a final concentration of 0.2 µM. 100 µl of each condition was added to a plate well, before a reaction was initiated with 100 µl of 50 mM urea. Giving final concentrations of urease (1.5 µM), pyranine (0.25 µM), urea (25 mM) and 0.1 µM CCCP/valinomycin/both/none in 200 µl with 1% ethanol. The fluorescent intensity was measured every 0.5 mins using the plate reader. A fluorescence intensity ratio (450/405 nm) of pyranine was calculated before being converted into pH using HPTS calibration curve. pH was plotted over time before being fitted with Hill1 to extract clock time (k) and the percentage change in clock time from the 1% ethanol control was determined (Fig 2). Three independent repeats were performed and ± standard error was used to present error. Extracted clock time values were analysed using one-way ANOVA, followed by Tukey's multiple comparisons test where a p-value > 0.05 was considered not significant. No significant difference between the control clock time and any condition was found, so we can be confident the increased clock time in the experiments was due to a change in membrane permeability.

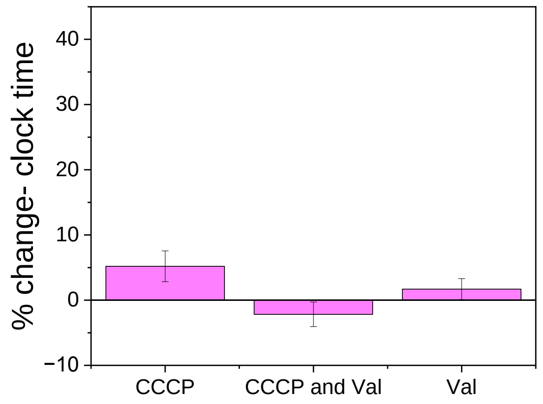


**Figure S3.** Percentage change in clock time between urease reaction in bulk (urease 1.5 µM, pyranine 0.25 µM, urea 25 mM) in 1% ethanol and either CCCP (0.1 µM), valinomycin (0.1 µM) or CCCP and valinomycin (both 0.1 µM). (n=3, error = ±SE). Comparison of the mean were analysed using one-way ANOVA followed by Tukey's multiple comparisons test with a p-value > 0.05, considered not significant.

# 3. Modelling of the urea-urease reaction in liposomes

The system was modelled using a set of coupled ordinary differential equations (ODEs) that describe the rate of change of species within the solution. The main processes in the model were (3.1) the enzyme-catalysed reaction in the lipid vesicles, (3.2) the equilibria that govern the pH in the vesicles and the surrounding solution and (3.3) the mass transfer of molecules between the vesicles and external solution.

## 3.1. Urease-catalysed reaction

Urease-catalysed hydrolysis of urea yields ammonia and carbon dioxide:

R1 $$CO(NH_2)_2 + H_2O \xrightarrow{urease} 2NH_3 + CO_2$$

The rate of the enzyme-catalysed reaction was given by a modified Michaelis-Menten equation (for simplicity free acid is included as $H^+$ rather than $H_3O^+$):[4,5]

$$v_0 = \frac{R_{max}[\text{urea}]}{(K_M + [\text{urea}]\left(1 + \frac{[\text{urea}]}{K_S}\right)\left(1 + \frac{K_{es2}}{[\text{H}^+]} + \frac{[\text{H}^+]}{K_{es1}}\right)\left(1 + \frac{[\text{NH}_4{}^+]}{K_P}\right)} \quad \text{(SE9)}$$

where $R_{max} = k_1[E]_0$ and $k_1$ is the rate constant for irreversible decomposition of the enzyme-substrate complex into products, $[E]_0$ is the total concentration of enzyme (U/ml), $K_M$ is the Michaelis constant, $K_{es2}$ and $K_{es1}$ are the protonation equilibria of the substrate-enzyme complex that give rise to the bell-shaped rate-pH curve. Substrate and product inhibition terms were included: $K_S$ = equilibrium constant for uncompetitive substrate inhibition and $K_P$ = equilibrium constant for non-competitive product inhibition.

The turnover number ($k_{cat}$ ($s^{-1}$)) of urease varies depending on the source, purity and conditions of the assay.[4] Turnover numbers for Jack Bean urease are reported as $k_{cat} = (1 – 90) \times 10^3\ s^{-1}$ (pH 5.5 - 8 and 15 - 38 ℃).[6–8] In our earlier work, $k_I$ was defined as $k_{cat}/p/M_r$, such that $k_{cat}[E]= k_I[E]_0$ where [E] is expressed in molar concentration and $[E]_0$ is in U/ml. Here, $p$ represents the activity of pure urease (600 U/mg where 1 unit (U) = 1 µmol $NH_3$ $min^{-1}$ at pH 7 and 25 °C) and $M_r$ is the molecular mass of Jack Bean urease (545000 g $mol^{-1}$). Using $k_{cat}$ of $1.2 \times 10^3\ s^{-1}$, we previously determined $k_I = 2.2 \times 10^{-4}$ M $U^{-1}$ ml $min^{-1}$.[9,10] Given uncertainties in enzyme activity in the vesicles, $R_{max}$ was used to fit the clock reaction time in the standard case (Fig. 3A) and fixed for the rest of the analysis. We used $R_{max}$ = 0.066 M/ min, giving $[E]_0$ = 300 U/ ml, and comparing well with experimental estimation of maximum activity (330 U/ mL).

## 3.2. pH equilibria

The pH inside and outside the liposomes is determined by the following reversible reactions:

| | | | |
|---|---|---|---|
| R2 | $NH_4^+ \rightleftharpoons NH_3 + H^+$ | $pK_a = 9.25$ | rate = $k_2[NH_4^+] – k_{2r}[NH_3][H^+]$ |
| R3 | $CO_2 + H_2O \rightleftharpoons H^+ + HCO_3^-$ | $pK_a = 6.35$ | rate = $k_3[CO_2] – k_{3r}[HCO_3^-][H^+]$ |

| | | | |
|---|---|---|---|
| R4 | $HCO_3^- \rightleftharpoons H^+ + CO_3^{2-}$ | p$K_a$ = 10.25 | rate = $k_4[HCO_3^-] - k_{4r}[CO_3^{2-}][H^+]$ |
| R5 | $H_2O \rightleftharpoons H^+ + OH^-$ | p$K_a$ = 14 | rate = $k_5[H_2O] - k_{5r}[OH^-][H^+]$ |
| R6 | $PyOH^{3-} \rightleftharpoons PyO^{4-} + H^+$ | p$K_a$ = 7.39 | rate = $k_6[PyOH^{3-}] - k_{6r}[PyO^{4-}][H^+]$ |
| R7 | $CH_3COOH \rightleftharpoons CH_3COO^- + H^+$ | p$K_a$ = 4.79 | rate = $k_7[CH_3COOH] - k_{7r}[CH_3COO^-][H^+]$ |

The acid equilibria rate constants are well established.[11,12] The pyranine p$K_a$ varies depending on the ionic strength.[13]

## 3.3. Transfer rates and permeability

### 3.3.1. The transfer of neutral species

As with our previous work,[10] the transfer of neutral species across the membrane boundary was assumed to follow a simple solubility-diffusion mechanism. The flux of a solute species $i$ is given by:

$$J_i = -P_i \Delta C \quad \text{(SE10)}$$

where $J_i$ is the flux, $P_i$ is the permeability coefficient (cm/min) that depends on the nature of the membrane and the chemical species, and $\Delta C$ is the transmembrane concentration gradient of solute species $i$ across a membrane of thickness $L$. For a spherical vesicle with surface area $A$ and volume $V$, the rate of change in concentration is expressed as:[12]

$$\frac{dC}{dt} = \frac{A}{V} P_i \, \Delta C = \frac{3P_i}{r} \Delta C \quad \text{(SE11)}$$

The permeability coefficients reported in the literature vary depending on the lipid properties and determination method and may be affected by unstirred layers.[14] We set the permeability values for the permeability coefficients as (cm/ min): $P_{CO2} = 20$, $P_{NH3} = 2$, $P_{urea} = 8.5 \times 10^{-5}$, $P_{AA} = 0.6$, $P_{Cl-} = 6 \times 10^{-7}$, $P_{K+} = 2 \times 10^{-10}$, $P_{Na+} = 2 \times 10^{-10}$, $P_{H+} = 1 \times 10^{-3}$ for 18:1 DOPC lipid membranes to be broadly consistent with literature values.[15-17] Permeability values for different membrane lengths (14:1 and 16:1 POPC membranes) are discussed below and stated in Table S1.[15]

### 3.3.2. The transfer of ionic species

For the passive transport of ions across the vesicle membrane, the incorporation of both a membrane capacitance and membrane potential governed by net number of ions crossing the membrane into the lumen of the vesicle is required. Capacitance, in the context of cell membranes and the build-up of membrane potential, reflects the membrane's ability to store electrical charge, with a greater capacitance allowing for more ions to pass before charge build up halts the process.

The flux of ion $n$ through the membrane is given by:[18-21]

$$J_n = -P_n \left( \frac{C_n^{in} - C_n^{out} \, e^{-U}}{1 - e^{-U}} \right) \quad \text{(SE12)}$$

where $P_n$ is the permeability coefficient (cm/min) of ion $n$ across the membrane, $\boldsymbol{C_n^{in}}$ and $\boldsymbol{C_n^{out}}$ are the concentration of ion $n$ inside and outside respectively, and $U$ is the reduced membrane potential, $\Delta\psi/k_B T$. Here, $k_B T$ is the thermal energy unit where $k_B$ is the Boltzmann constant and T is the temperature measured in Kelvin, and $\Delta\psi$ is the membrane potential arising from the net charge accumulated in the liposome using a capacitor model for the membrane.

The membrane potential is defined in Equation SE13:

$$\Delta\Psi = \frac{F\,V}{C_0\,S}\left(n\sum_n z_n\,C_n^{enter}\right) \tag{SE13}$$

where, $F$ is Faraday's Constant, $V$ is the liposome volume, $C_0$ is the membrane capacitance, $z_n$ is the charge (valency) on ion $n$ and $C_n^{enter}$ is the concentration of ions that have entered the lumen. The rate of concentration change for a permeating ion in a spherical liposome is:

$$\frac{dC}{dt} = -\frac{P_n\,S\,z\,U}{V}\left(\frac{C_n^{in} - C_n^{out}e^{-zU}}{1 - e^{-zU}}\right) \tag{SE14}$$

For a detailed derivation of these equations, please refer to Cellular Biophysics by T. F. Weiss.[18] The membrane capacitance values are taken as 0.8 µF/ cm² for 18:1 DOPC membranes (for other membrane lengths, see discussion below and Table S1). In this study, we considered the permeabilities of $H^+$, $K^+$, $Na^+$ and $Cl^-$.

In experiments, proton permeability was enhanced with the addition of the protonophore carbonyl cyanide m-chlorophenyl hydrazone (CCCP), which electrically catalyses the transport of protons across membranes. Proton permeability is a topic of ongoing debate, with reported values ranging widely from $10^{-10}$ – $10^{-2}$ m/min.[22] This variability likely stems from a rapid initial proton transport component that quickly balances with the formation of an electrical gradient, making it highly dependent on experimental conditions, as well as lipid type. Before the addition of CCCP, we assumed the permeability for $H^+$ to be $1 \times 10^{-3}$ cm/min, increasing to $3 \times 10^{-1}$ cm/ min after CCCP addition, based on literature on CCCP mechanisms.[23-24] Valinomycin facilitates the rapid translocation of potassium and ammonium across the bilayer. The permeability for $K^+$ was assumed to be $2 \times 10^{-10}$ cm/min prior to valinomycin addition and set to $3 \times 10^{-8}$ cm/ min post-addition.[19,20] Given valinomycin's higher affinity for $K^+$ than $NH_4^+$, we assumed no permeability for ammonium in the presence of potassium.

### 3.3.4. Variation according to membrane length

The compositions 18:1, 16:1, and 14:1 lipid membranes have decreasing membrane bilayer thicknesses, measured as 27 Å, 23.5 Å and 20 Å, respectively, for monounsaturated phosphatidylcholine (PC) membranes.[25] Permeabilities for urea, $H^+$ and $K^+$ in these membranes were given by Paula et al.[15] In the absence of data for the permeabilities with the specific membranes used here, we increased permeability coefficients by factor 1.5 for neutral/anion and factor of 100 for cations for C = 16, and for C = 14, the permeability coefficients were increased by factor 2 for neutral/anion and factor of 800 for cations, in line with literature data for similar membranes.[26-29] Membrane capacitance decreases with membrane thickness according to Salipante et al.;[30] however, under the conditions explored here changes in $C_0$ had limited effect on the clock time hence it was fixed at 0.8 µF/ cm².

**Table S1.** Permeability and membrane capacitance values used in simulations for 14:1, 16:1 and 18:1 PC vesicles

| Chain length (carbon number) | $P_{urea}$ cm/min | $P_{H+}$ cm/min | $P_{NH3}$ cm/min | $P_{CO2}$ cm/min | $P_{AA}$ cm/min | $P_{Na}$ cm/min | $P_{Cl-}$ cm/min |
|---|---|---|---|---|---|---|---|
| 14 | $1.7 \times 10^{-4}$ | $8.0 \times 10^{-1}$ | $4.0 x 10^{0}$ | $4.0 \times 10^{1}$ | $1.2 \times 10^{0}$ | $1.6 \times 10^{-7}$ | $1.2\times 10^{-6}$ |
| 16 | $1.3 \times 10^{-4}$ | $1.0 \times 10^{-1}$ | $3.0 \times 10^{0}$ | $3.0 \times 10^{1}$ | $9.0 \times 10^{-1}$ | $2.0 \times 10^{-8}$ | $9.0\times 10^{-7}$ |
| 18 | $8.5 \times 10^{-5}$ | $1.0 \times 10^{-3}$ | $2.0 \times 10^{0}$ | $2.0 \times 10^{1}$ | $6.0 \times 10^{-1}$ | $2.0 \times 10^{-10}$ | $6.0\times 10^{-7}$ |

## 3.4. Model equations and parameters

The ODE model considers all the 13 chemical species in reactions R1–R7 (not including water) to produce coupled rate equations, including the pH determining equilibria and the modified Michaelis–Menten enzyme rate. The values of all the rate constants taken in this work are shown in Table S2. The rate equations were solved using MATLAB with integration method ode15s.

The initial concentrations, unless otherwise stated, were taken from the experimental conditions: in the vesicles, [pyranine] = 50 µm, $[H^+] = 1 \times 10^{-5}$ M, $[OH^-] = 1 \times 10^{-9}$ M, [acetate buffer] = 50 mM ([acetate] = 32 mM, [acetic acid] = 18 mM), $[Na^+]$ = 120 mM (from NaCl to give 100 mM ionic strength buffer, sodium acetate and sodium dihydrogen phosphate from type III enzyme powder – see section 1.1), $[Cl^-]$ = 70 mM (from NaCl). The solution of vesicles in buffer was mixed with an equal amount of urea solution to give final concentrations in the external solution of: [urea] = 25 mM, $[H^+] = 1 \times 10^{-5}$ M, [acetate buffer] = 25 mM ([acetate] = 16 mM, [acetic acid] = 9 mM); $[Na^+]$ = 50 mM (from NaCl to give 100 mM ionic strength buffer and sodium acetate), $[Cl^-]$ = 35 mM (from NaCl). All other initial concentrations were set to zero.

**Table S2.** Rate constants (20°C) and enzyme constants for the urease system in an 18:1 DOPC vesicle.

| | | | | | | |
|---|---|---|---|---|---|---|
| Equilibria rate constants | $k_2$ min$^{-1}$ | $k_{2r}$ M$^{-1}$ min$^{-1}$ | $k_3$ min$^{-1}$ | $k_{3r}$ M$^{-1}$ min$^{-1}$ | $k_4$ min$^{-1}$ | $k_{4r}$ M$^{-1}$ min$^{-1}$ |
| | 1440 | $2.58 \times 10^{12}$ | 2.22 | $4.74 \times 10^{6}$ | 168 | $3 \times 10^{12}$ |
| | $k_5$ M min$^{-1}$ | $k_{5r}$ M$^{-1}$ min$^{-1}$ | $k_6$ min$^{-1}$ | $k_{6r}$ M$^{-1}$ min$^{-1}$ | $k_7$ (acetate buffer) (MES buffer) min$^{-1}$ | $k_{7r}$ M$^{-1}$ min$^{-1}$ |
| | $6 \times 10^{-2}$ | $6 \times 10^{12}$ | 60 | $1.5 \times 10^{9}$ | $4.68 \times 10^{7}$ $1.9 \times 10^{6}$ | $2.7 \times 10^{12}$ |
| Urease Enzyme constants | $R_{max}$ M min$^{-1}$ | $K_M$ M | $K_{es1}$ | $K_{es2}$ | $K_s$ M | $K_p$ M |
| | 0.066 | $3 \times 10^{-3}$ | $5 \times 10^{-6}$ | $2 \times 10^{-9}$ | 3 | 0.2 |
| Transport constants | $P_{urea}$ cm/min | $P_{NH3}$ cm/min | $P_{CO2}$ cm/min | $P_{AA}$ cm/min | | |
| | $8.5 \times 10^{-5}$ | 2 | $2 \times 10^{2}$ | $6 \times 10^{-1}$ | | |
| Ion Transport constants | $P_{H+}$ cm/min | $P_{H+}$ CCCP cm/min | $P_{K+}$ cm/min | $P_{K+}$ Val cm/min | $P_{Na+}$ cm/min | $P_{Cl-}$ cm/min |
| | $1 \times 10^{-3}$ | $3 \times 10^{-2}$ | $2 \times 10^{-10}$ | $6 \times 10^{-8}$ | $2 \times 10^{-10}$ | $6 \times 10^{-7}$ |
| Other constants | Vesicle radius m | Bilayer capacitance per unit area ($C_0$) µF cm$^{-2}$ | | $k_BT/e$ mV | Avogadro's number ($N_A$) mol$^{-1}$ | Volume fraction, $\phi = NV_i / V_o$ |
| | $8.0 \times 10^{-8}$ | 0.8 | | 25.69 | $6.022 x 10^{23}$ | 0.002 |

Inside the vesicles, the rate of change of the concentration of a species $A_i$ is determined by the reaction rate and the net transfer rate (where applicable):

$$\frac{dA_i}{dt} = f(A_i) + \varphi \tag{SE15}$$

where $f(A_i)$ contains all the relevant reaction terms and $\varphi$ represents the net transfer rate of species across the membrane. For example, for neutral species this net transfer rate is equal to:

$$\varphi = \frac{3P_i}{r}(A_o - A_i) \tag{SE16}$$

where $P_i$ is the permeability coefficient, $r$ is the radius of the liposome and $A_o$ is the concentration in the outer solution.

For identical liposomes, the rate of change of concentration of each species in the surrounding solution includes an additional term, called the vesicle volume fraction, $\phi$. This is equal to $\phi = N\, V_i \,/\, V_o$, where $N$ represents the number of vesicles, $V_i$ is the internal volume of an individual vesicle, and $V_o$ is the external volume. This represents the ratio of the total volume of vesicles to the total volume of solution. Consequently, the rate of change of the concentration of a species $A$ outside the vesicles is

$$\frac{dA_o}{dt} = f(A_o) + \phi\, \varphi \tag{SE17}$$

For example, the rate of change of concentration of each species in the surrounding solution for neutral molecules that can permeate the vesicle membrane is:

$$\frac{dA_o}{dt} = f(A_o) + \phi\, \frac{3P_i}{r}(A_o - A_i) \tag{SE18}$$

## 3.5. Model assumptions

The following assumptions underpin our modeling approach:

- Vesicle characteristics: We assume the vesicles in our model to be 100% unilamellar and monodisperse, with a uniform size of 162 nm in diameter. Furthermore, we assume a uniform distribution of enzymes within the vesicles.
- Reaction conditions and solution mixing: Temperature fluctuations are considered negligible. Both internal and external solutions are assumed to undergo instantaneous and uniform mixing.
- Transport of other cations and anions has negligible effect on the trends reported here.

## References


1. van Vliet, A. H., Kuipers, E. J., Waidner, B., Davies, B. J., de Vries, N., Penn, C. W., Vandenbroucke-Grauls, C. M., Kist, M., Bereswill, S., & Kusters, J. G. Nickel-responsive induction of urease expression in Helicobacter pylori is mediated at the transcriptional level. *Infection and immunity,* **2001,** *69* (8), 4891–4897. DOI: 10.1128/IAI.69.8.4891-4897.2001
2. Mozafari, M.R.; Mazaheri, E.; Dormiani, K. Simple Equations Pertaining to the Particle Number and Surface Area of Metallic, Polymeric, Lipidic and Vesicular Nanocarriers. *Sci. Pharm.* **2021**, *89*, 15. DOI: 10.3390/scipharm89020015
3. Ruysschaert, T., A. Marque, J. L. Duteyrat, S. Lesieur, M. Winterhalter, and D. Fournier. Liposome retention in size exclusion chromatography. *BMC Biotechnol.* **2005** *5* (11)*,* 1–13. DOI: 10.1186/1472-6750-5-11
4. Krajewska, B.; Ciurli, S. Jack bean (Canavalia ensiformis) urease. Probing acid–base groups of the active site by pH variation. *Plant Physiol. Biochem.* **2005**, *43*, 651-658. DOI: 10.1016/j.plaphy.2005.05.009
5. Krajewska, B. Ureases I. Functional, catalytic and kinetic properties: A review. *Journal of Molecular Catalysis B: Enzymatic*, **2009**, *59* (1-3), 9–21. DOI: 10.1016/j.molcatb.2009.01.003
6. Krajewska, B., van Eldik, R. & Brindell, M. Temperature- and pressure-dependent stopped-flow kinetic studies of jack bean urease. Implications for the catalytic mechanism. *J Biol Inorg Chem 17*, **2012**, 1123–1134. DOI: 10.1007/s00775-012-0926-8
7. Lopreore, C., & Byers, L. D. The urease-catalyzed hydrolysis of thiourea and thioacetamide. *Archives of biochemistry and biophysics*, **1998**, *349* (2), 299–303. DOI: 10.1006/abbi.1997.0477
8. Dixon, N. E., Riddles, P. W., Gazzola, C., Blakeley, R. L., & Zerner, B. Jack bean urease (EC 3.5.1.5). V. On the mechanism of action of urease on urea, formamide, acetamide, N-methylurea, and related compounds. *Canadian journal of biochemistry*, **1980**, *58* (12), 1335–1344. DOI: 10.1139/o80-181
9. Hu, G.; Pojman, J. A.; Scott, S. K.; Wrobel, M. M.; Taylor, A. F. Base-Catalyzed Feedback in the Urea - Urease Reaction. *J. Phys. Chem. B* **2010**, *114* (44), 14059-14063. DOI: 10.1021/jp106532d
10. Miele, Y.; Jones, S. J.; Rossi, F.; Beales, P. A.; Taylor, A. F. Collective Behavior of Urease PH Clocks in Nano- and Microvesicles Controlled by Fast Ammonia Transport. *J. Phys. Chem. Lett.* **2022**, *13* (8), 1979–1984. DOI: 10.1021/acs.jpclett.2c00069
11. Wang, X., Conway, W., Burns, R., McCann, N. & Maeder, M. Comprehensive study of the hydration and dehydration reactions of carbon dioxide in aqueous solution. *Journal of Physical Chemistry A, **2010**, 114*, 1734–1740. DOI: 10.1021/jp909019u
12. Eigen, M. Proton Transfer, Acid-Base Catalysis, and Enzymatic Hydrolysis. Part I: ELEMENTARY PROCESSES. *Angewandte Chemie International Edition in English* 3, **1964**, 1–19. DOI: 10.1002/anie.196400011
13. Avnir, Y. & Barenholz, Y. pH determination by pyranine: Medium-related artifacts and their correction. *Analytical Biochemistry*, **2005**, 347(1), 34-41 DOI: 10.1016/j.ab.2005.09.026.
14. Missner, A. & Pohl, P. 10 Years of the Meyer-Overton Rule: Predicting Membrane Permeability of Gases and Other Small Compounds. *ChemPhysChem*, **2009**, *10*, 1405–1414 DOI: 10.1002/cphc.200900270.
15. Paula, S., Volkov, A. G., Van Hoek, A. N., Haines, T. H. & Deamer, D. W. Permeation of Protons, Potassium Ions, and Small Polar Molecules Through Phospholipid Bilayers as a Function of Membrane Thickness. *Biophysical Journal, **1996**, 70*, 339–348. DOI: 10.1016/S0006-3495(96)79575-9

16. Lande, M. B., Donovan, J. M. & Zeidel, M. L. The relationship between membrane fluidity and permeabilities to water, solutes, ammonia, and protons. *J Gen Physiol.,* **1995,** *106*, 67–84. DOI: 10.1085/jgp.106.1.67
17. Xiang, T. X. & Anderson, B. D. Permeability of Acetic Acid Across Gel and Liquid-Crystalline Lipid Bilayers Conforms to Free-Surface-Area Theory. *Biophys J,* **1997**, *72*, 223–237. DOI: 10.1016/S0006-3495(97)78661-2
18. Weiss, T. F. *Cellular Biophysics. Vol. 1 Transport*; MIT Press, 1996.
19. Veshaguri, S.; Christensen, S. M.; Kemmer, G. C.; Møller, M. P.; Lohr, C.; Christensen, A. L.; Bo, H.; Jørgensen, I. L.; Schiller, J.; Hatzakis, N. S.; Grabe, M.; Pomorski, T. G.; Stamou, D. Direct Observation of Proton Pumping by a Eukaryotic P-Type ATPase. **2016**, *351* (6280), 1469–1473. DOI: 10.1126/science.aad6429
20. Singh, A.; Marcoline, F. V.; Veshaguri, S.; Kao, A. W.; Bruchez, M.; Mindell, J. A.; Stamou, D.; Grabe, M. Protons in Small Spaces: Discrete Simulations of Vesicle Acidification. *PLoS Comput. Biol.* **2019**, *15* (12), 1–21. DOI: 10.1371/journal.pcbi.1007539
21. Ishida, Y.; Nayak, S.; Mindell, J. A.; Grabe, M. A Model of Lysosomal PH Regulation. *J. Gen. Physiol.* **2013**, *141* (6), 705–720. DOI: 10.1085/jgp.201210930
22. Decoursey, T. E. Voltage-gated proton channels and other proton transfer pathways. *Physiological Reviews, **2003**, 83*, 475–579. DOI: 10.1152/physrev.00028.2002
23. Kasianowicz, J., Benz, R., & McLaughlin, S. The kinetic mechanism by which CCCP (carbonyl cyanidem-Chlorophenylhydrazone) transports protons across membranes. *The Journal of Membrane Biology*, **1984**, *82* (2), 179–190. DOI: 10.1007/bf01868942
24. Fuks, B., & Homble, F. Mechanism of Proton Permeation through Chloroplast Lipid Membranes. *Plant Physiology*, **1996**, *112* (2), 759–766. DOI: 10.1104/pp.112.2.759
25. Lewis, B. A.; Engelman, D. M. Lipid Bilayer Thickness Varies Linearly with Acyl Chain Length in Fluid Phosphatidylcholine Vesicles. *J. Mol. Biol.* **1983**, *166* (2), 211–217. DOI: 10.1016/S0022-2836(83)80007-2
26. Hannesschlaeger, C.; Horner, A.; Pohl, P. Intrinsic Membrane Permeability to Small Molecules. *Chemical Reviews*. **2019**, *119* (9), 5922–5953. DOI: 10.1021/acs.chemrev.8b00560
27. Frallicciardi, J.; Melcr, J.; Siginou, P.; Marrink, S. J.; Poolman, B. Membrane Thickness, Lipid Phase and Sterol Type Are Determining Factors in the Permeability of Membranes to Small Solutes. *Nature Communications.* **2022**, *13* (1), 1605. 1-12. DOI: 10.1038/s41467-022-29272-x
28. Paula, S.; Volkov, A. G.; Deamer, D. W. Permeation of Halide Anions through Phospholipid Bilayers Occurs by the Solubility-Diffusion Mechanism. *Biophysical Journal*. **1998**, *74* (1), 319–327. DOI: 10.1016/s0006-3495(98)77789-6
29. Megens, M,; Korman, C. E.; Ajo-Franklin, C. M.; Horsley, D. A. Faster-Than-Anticipated Na+/Cl− Diffusion across Lipid Bilayers in Vesicles. *Biochimica et Biophysica Acta (BBA) – Biomembranes.* **2014**, *1838* (10), 2420–2424. DOI: 10.1016/j.bbamem.2014.05.010
30. Salipante, P. F.; Knorr, R. L.; Dimova, R.; Vlahovska, P. M. Electrodeformation Method for Measuring the Capacitance of Bilayer Membranes. *Soft Matter* **2012**, *8* (14), 3810–3816. DOI: 10.1039/c2sm07105c
